# Decomposing the modulation of interactions between neuronal populations

**DOI:** 10.64898/2026.05.14.725145

**Published:** 2026-05-18

**Authors:** Marco Celotto, J. Samuel Sooter, Kyle R. Jenks, Sofie Ährlund-Richter, Mriganka Sur, Stefano Panzeri

**Affiliations:** 1 Picower Institute for Learning and Memory, Massachusetts Institute of Technology, Cambridge, MA, USA; 2 Institute for Neural Information Processing, Center for Molecular Neurobiology (ZMNH), University Medical Center Hamburg-Eppendorf (UKE), Hamburg, Germany; 3 Department of Brain and Cognitive Sciences, Massachusetts Institute of Technology, Cambridge, MA, USA; 4 UA Integrative Systems Neuroscience Group, Department of Physics, University of Arkansas, Fayetteville, AR, USA

## Abstract

Identifying subpopulations of neurons that interact with each other from simultaneous recordings of populations of many neurons is key for understanding across-brain communication with cellular resolution. Recent work identified communication subspaces, which capture additive interactions between pairs of high-dimensional neural populations through a small number of source and target activity patterns. However, no current method captures how a third, potentially multivariate variable - such as behavioral state or the activity of a third population - modulates these interactions. Here we extend the communication subspace framework by parameterizing modulation as a low-rank tensor. This identifies multiplicative interaction channels (MICs), defined as triplets of source, target, and modulator activity patterns, in which the modulator pattern gates the source-target interaction. We derive MICs as a bilinear perturbation of reduced-rank regression. We develop a hierarchical fitting pipeline and provide a closed-form decomposition that quantifies whether modulation reshapes the modulator-averaged baseline interaction, recruits private dimensions of one population, or opens new interactions. In simulations, MICs reliably recover the presence and geometry of ground-truth modulation even in the high-dimensional, low-sample regime. Applying MICs to simultaneous calcium imaging of prefrontal axons and interneurons in the visual cortex revealed that behavioral state asymmetrically modulates top-down interactions, reconfiguring the patterns of prefrontal projections that interact with a stable set of visual interneuron activity patterns. By providing an efficient and compact characterization of modulatory interactions, MICs enable asking new questions about how potentially high-dimensional variables shape interactions between neural populations.

## 1 Introduction

Brain computations supporting cognitive functions arise from interactions between multiple brain regions [1, 2]. It is becoming clear that inter-area interactions are implemented in the brain with very fine cellular-level resolution [3–5], at the level of specific subpopulations of neurons dynamically interacting with other subpopulations. As modern recording technologies access hundreds to thousands of neurons across multiple regions [6–8], a fundamental computational challenge is to develop analysis tools that can map these interactions from simultaneous recordings of many neurons and work even in the under-sampled regime typical of brain data [9]. Classical pairwise approaches - correlation [10], Granger causality [11, 12], and information theory [13, 14] - were developed for scalar variables or small populations, and they do not scale up with the number of recorded units, making them impractical for high-dimensional recordings [15].

Reduced-rank regression (RRR) to identify *communication subspaces* [16] has emerged as a powerful framework to address this challenge. Constraining the regression matrix relating two populations to be low-rank identifies low-dimensional patterns of activity of one population that co-vary with low-dimensional activity patterns of the other population, while excluding variability private to each population [17]. The low-rank constraint dramatically reduces the number of free parameters, achieving data-efficiency and scalability. Communication subspaces have become a standard tool to study inter-areal brain interactions with cellular resolution [18–22].

However, interactions between neural populations are not static. They can change with cognitive state, movement parameters, task context, and the activity of other brain regions. For example, attention *modulates* top-down interactions between higher cortical areas and sensory cortex [23, 24], yet the population-level structure of these modulations remains poorly understood. Modulation effects are usually modelled as multiplicative - rather than additive - effects of a modulator on the relationship between other variables [25–28]. Importantly, modulatory variables can themselves be high-dimensional, such as multiple movement parameters [29] or the activity of other modulatory populations [30]. Despite the importance of modulatory interactions, communication subspace analysis is designed to capture pairwise, additive relationships, but not how a third, potentially high-dimensional variable multiplicatively reshapes the interaction between two populations.

Here, we introduce Multiplicative Interactions Channels (*MICs*), a method extending communication subspaces to capture how a high-dimensional modulator may reshape interactions between two populations (a source and a target of interactions). We model the modulator-induced perturbation of the source-target mapping as a low-rank three-way tensor and parameterize it via a Canonical Polyadic (CP) decomposition [31–33]. Low-rank tensor decompositions can be robustly applied to neural population data [34–36] and to regression with multivariate covariates [37–39]. Building on this work, we parameterize the perturbation as a sum of rank-1 *multiplicative interaction channels*, each pairing one modulator pattern with the source-target interaction pattern it gates. We mathematically derive the MICs model, describe a hierarchical data-fitting procedure with cross-validated rank and regularization selection, and show that the geometry of each MIC relative to the modulator-averaged, baseline communication subspace can be decomposed into four interpretable components. We next validate MIC on simulated data. We finally apply it to simultaneous recordings of top-down prefrontal axonal projections and visual cortical interneurons in mice, finding that the behavioral state changes the top-down projection patterns that interact with a stable set of visual neuron activity patterns.

## 2 Mathematical derivation of multiplicative interaction channels

### 2.1 Background: communication subspaces via reduced-rank regression

We consider simultaneous observations of three possibly high-dimensional variables: a *source* $x\in {\mathbb{R}}^{n_{X}}$, a *target* $y\in {\mathbb{R}}^{n_{Y}}$, and a *modulator* $z\in {\mathbb{R}}^{n_{Z}}$. For example, x and y could represent the activity of two neural populations, and z could be a set of behavioral variables (e.g., running speed, pupil diameter), or the activity of a third modulatory population. Given N simultaneous observations, such as time points in a multivariate time series, we build data matrices $X\in {\mathbb{R}}^{N\times n_{X}}$, $Y\in {\mathbb{R}}^{N\times n_{Y}}$, and $Z\in {\mathbb{R}}^{N\times n_{Z}}$. The communication subspace framework [16] identifies components of source activity linearly related to other specific components of target activity. It models the target as a low-rank linear function of the source:

(1) $$Y=XA+E,\;\text{rank}(A)=m_{A},$$

where $A\in {\mathbb{R}}^{n_{X}\times n_{Y}}$ and E denotes residual variability. The rank constraint implies that A can be factorized as $A=U_{A}V_{A}^{⊤}$, with $U_{A}\in {\mathbb{R}}^{n_{X}\times m_{A}}$ and $V_{A}\in {\mathbb{R}}^{n_{Y}\times m_{A}}$. The columns of $U_{A}$ and $V_{A}$ define, respectively, the $m_{A}$ source and target patterns that co-vary with each other; patterns outside $\text{col}\left(U_{A}\right)$ and $\text{col}\left(V_{A}\right)$ are private to each population [16]. The optimal A is obtained by ridge-regularized reduced-rank regression (ridge-RRR) [40, 17] (Appendix A.1). We refer to $\text{col}\left(U_{A}\right)$ and $\text{col}\left(V_{A}\right)$ as the source and target *additive interaction subspaces* (AIS), rather than “communication subspaces” [16], as RRR at zero lag captures additive co-variation, not directed transmission [11, 12, 17], and source/target swapping does not, on its own, establish directionality [18].

### 2.2 Beyond AIS: modeling low-dimensional multiplicative interactions

**Figure 1:**
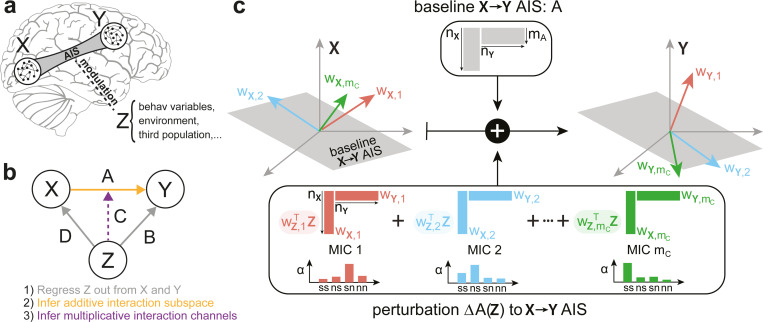
Sketch of MICs. (a) MICs quantify how the AIS between a multivariate source X and target Y changes as a function of a modulator Z. (b) Schematic of the modulation-channel fitting pipeline. (c) The perturbation ΔA(z) to the baseline X→Y mapping A is parameterized as a low-rank tensor: low-dimensional components of X and Z interact bilinearly to drive specific activity patterns in Y. Each channel’s source and target directions need not lie within the baseline AIS; their geometry relative to it is summarized by four indices ($\alpha_{ss}$, $\alpha_{ns}$, $\alpha_{sn}$, $\alpha_{nn}$; see Sec. 2.4).

AIS capture *additive*, linear relationships between two variables. However, many interactions in neuroscience are hypothesized to be *modulated* by additional variables. We thus ask how the modulator Z changes the linear mapping from source to target (Fig. 1a). We model the instantaneous effective interaction operator as a z-dependent perturbation of the baseline mapping:

(2) $$A_{\mathrm{eff}}(z)=A+\text{Δ}A(z),$$

where A is the baseline AIS and ΔA(z) captures the z-dependent modulation. We assume ΔA(z) to be linear in z, so that its action on the source is bilinear in x and z. We thus parameterize ΔA(z) as a sum of $m_{C}$ rank-1 *multiplicative interaction channels*^1^:

(3) $$\Delta A(z)=\sum_{i=1}^{m_{C}}\left(w_{Z,i}^{⊤}z\right)\;w_{X,i}w_{Y,i}^{⊤},$$

where for each channel i, the weight vectors $w_{X,i}\in {\mathbb{R}}^{n_{X}}$, $w_{Y,i}\in {\mathbb{R}}^{n_{Y}}$, and $w_{Z,i}\in {\mathbb{R}}^{n_{Z}}$ define source, target, and modulator axes, respectively. Intuitively, MIC i captures the fact that when the modulator aligns with $w_{Z,i}$ (i.e., the $w_{Z,i}^{⊤}z$ *score* is large), the effective mapping from source to target is perturbed by the rank-1 component $w_{X,i}w_{Y,i}^{⊤}$, scaled proportionally to $w_{Z,i}^{⊤}z$. This is a bilinear (multiplicative) interaction between source and modulator, analogous to an interaction term in a regression model, but operating in the high-dimensional setting where source, target, and modulator may each be high-dimensional (see Appendix A.2).

In matrix form, defining $W_{X}$, $W_{Z}$, $W_{Y}$ as the factor matrices whose columns are $\left\{w_{X,i}\right\}$, $\left\{w_{Z,i}\right\}$, $\left\{w_{Y,i}\right\}$ respectively, and letting ⊙ denote the element-wise (Hadamard) product, the modulation term across all N observations can be written compactly as:

(4) $$Y_{\mathrm{mod}}=\left[\begin{array}{c} x_{1}^{⊤}\Delta A\left(z_{1}\right)\\ \vdots \\ x_{N}^{⊤}\Delta A\left(z_{N}\right) \end{array}\right]=\left[\left(XW_{X}\right)⊙\left(ZW_{Z}\right)\right]\;W_{Y}^{⊤},$$

where $XW_{X}\in {\mathbb{R}}^{N\times m_{C}}$ and $ZW_{Z}\in {\mathbb{R}}^{N\times m_{C}}$ are the source and modulator scores (projections onto the respective axes), and their element-wise product captures the bilinear interaction. Mathematically, the collection ${\left\{\left(w_{X,i},w_{Z,i},w_{Y,i}\right)\right\}}_{i=1}^{m_{C}}$ defines a rank- $m_{C}$ CP decomposition [31–33] of a three-way tensor $C\in {\mathbb{R}}^{n_{Y}\times n_{X}\times n_{Z}}$.

Beyond the baseline interaction A and the modulation tensor 𝒞, the modulator may also exert direct additive effects on the target and on the source. We capture these with two additional low-rank linear terms: $B\in {\mathbb{R}}^{n_{Z}\times n_{Y}}$ (rank $m_{B}$) for the direct effect Z→Y, and $D\in {\mathbb{R}}^{n_{Z}\times n_{X}}$ (rank $m_{D}$) for the effect Z→X. Letting $X^{\prime }=X-ZD$ denote the *residualized source* (the component of source variability that is not linearly predictable from the modulator), the complete model is:

(5) $$Y=\underset{\text{baseline}\;\text{AIS}}{\underset{︸}{X^{\prime }A}}+\underset{\begin{array}{c} \text{additive}\\ Z\to Y \end{array}}{\underset{︸}{ZB}}+\underset{\text{modulation}}{\underset{︸}{\left[\left(X^{\prime }W_{X}\right)⊙\left(ZW_{Z}\right)\right]W_{Y}^{⊤}}}+E.$$

The residualized source $X^{\prime }$ enters both the baseline interaction and the modulation terms. The modulation term in Eq. 5 is a specific instance of CP-rank-constrained tensor-on-tensor regression [38], with the predictor restricted to the rank-1 outer product $x_{n}⊗z_{n}$ of two distinct observed variables. It is this restriction, rather than the CP structure of 𝒞 itself, that carries the bilinear-interaction form. The full MICs model (Eq. 5) embeds this term within an additive RRR framework with residualized source, a configuration not addressed in [38] (see Appendix A.2).

#### The baseline AIS operator A.

We assume all variables X, Y, and Z are zero-centered at the stage of model fitting (see Section 2.3). The expected value of the modulatory perturbation is thus $E[\Delta A(z)]=\sum_{i}E[w_{Z,i}^{⊤}z]\;w_{X,i}w_{Y,i}^{⊤}=0$. Thus, A represents the average effective AIS $E\left[A_{\text{eff}}(z)\right]=A$. MICs capture how each observation deviates from this average, depending on z.

### 2.3 Hierarchical model fitting and modulatory explained variance

Fitting all parameters of Eq. 5 jointly is computationally challenging due to the interaction between the additive and multiplicative terms. Instead, we adopt a hierarchical procedure that sequentially isolates each component (Algorithm 1; Appendix A.6; Fig. 1b).

#### Step 1: Regressing out additive effects of Z.

We first fit the additive effects of the modulator on both the target and the source via ridge-RRR:

(6) $$B=\mathrm{RRR}\left(Z,Y;m_{B},\lambda_{B}\right),\;D=\mathrm{RRR}\left(Z,X;m_{D},\lambda_{D}\right),$$

where RRR(⋅,⋅;m,λ) denotes ridge-RRR with rank m and penalty λ (see Appendix A.1), and compute residuals $X^{\prime }=X-ZD$, $Y^{\prime }=Y-ZB$. This removes variance in both X and Y that is linearly attributable to Z alone. Regressing Z out of X (via D) is particularly important: it ensures that downstream terms capture the X−Y relationship that is not confounded by Z independently driving both X and Y (see Sec. 3.1).

#### Step 2: Fitting baseline interaction.

Given the residuals, we fit the baseline interaction matrix as:

(7) $$A=\mathrm{RRR}\left(X^{\prime },Y^{\prime };m_{A},\lambda_{A}\right).$$

#### Step 3: Fitting MICs.

We fit the modulation tensor to the residuals of the baseline interaction model, $Y^{\prime \prime }=Y^{\prime }-X^{\prime }A$, by minimizing the regularized loss:

(8) $$ℒ\left(W_{X},W_{Z},W_{Y}\right)={\left\|Y^{\prime \prime }-\left[\left(X^{\prime }W_{X}\right)⊙\left(ZW_{Z}\right)\right]\;W_{Y}^{⊤}\right\|}_{F}^{2}+\lambda_{C}\|𝒞{\|}_{F}^{2},$$

where $\|𝒞{\|}_{F}^{2}=\mathrm{tr}\left(G_{Y}^{⊤}\left(G_{X}⊙G_{Z}\right)\right)$ is the squared Frobenius norm of the tensor expressed in terms of the Gram matrices $G_{X}=W_{X}^{⊤}W_{X}$, $G_{Z}=W_{Z}^{⊤}W_{Z}$, $G_{Y}=W_{Y}^{⊤}W_{Y}$ (see Appendix A.4). We minimize Eq. 8 via alternating least squares (ALS), cyclically updating each factor matrix while holding the other two fixed (Algorithm 2, [33, 38]). Each sub-problem reduces to a regularized linear system; closed-form update equations and computational details are provided in Appendix A.5.

#### Rank and regularization selection.

Each rank $\left(m_{A},m_{B},m_{C},m_{D}\right)$ and ridge penalty $\left(\lambda_{A},\lambda_{B},\lambda_{C},\lambda_{D}\right)$ is selected via K-fold cross-validation. For a given rank, the ridge penalty is optimized using Bayesian optimization [42]. The rank is selected as the smallest value whose cross-validated performance is within one SE of the maximum [16, 43]. The same cross-validation folds are used across all steps preventing information leakage between hierarchical stages (Algorithm 1).

#### Modulatory explained variance

Our primary measure of modulatory interaction strength is the *modulatory explained variance* $\left(\text{E}{\text{V}}_{\mathrm{mod}}\right)$, defined as the incremental cross-validated variance explained by the modulation term beyond the joint additive model. Denoting by ${\hat{Y}}_{\text{add}}^{\left(k\right)}={X_{\text{test}}^{\prime }}^{\left(k\right)}A^{\left(k\right)}+Z_{\text{test}}^{\left(k\right)}B^{\left(k\right)}$ the joint additive model prediction on the test set of fold k, and by ${\hat{Y}}_{\text{full}}^{\left(k\right)}={\hat{Y}}_{\text{add}}^{\left(k\right)}+\left[\left({X_{\text{test}}^{\prime }}^{\left(k\right)}W_{X}^{\left(k\right)})⊙(Z_{\text{test}}^{\left(k\right)}W_{Z}^{\left(k\right)}\right)\right]W_{Y}^{(k)⊤}$ the full model prediction:

(9) $${\text{EV}}_{\text{mod}}=\frac{1}{K}\sum_{k=1}^{K}\left[R_{\text{full}}^{2}(k)-R_{\text{add}}^{2}(k)\right],$$

where $R^{2}(k)$ is the coefficient of determination on fold k. A positive $\text{E}{\text{V}}_{\mathrm{mod}}$ indicates that the bilinear X−Z interaction explains target variance beyond the combination of additive terms. Analogously, EV_AIS_ is the incremental gain from adding $X_{\text{test}}^{\prime }(k)\;A^{\left(k\right)}$ over the modulator-only prediction $Z_{\text{test}}^{\left(k\right)}B^{\left(k\right)}$, yielding a variance partitioning of Y along the hierarchical fit.

### 2.4 Geometric decomposition of MICs

A MIC could modulate interaction geometry by rehaping baseline source-target interactions, engageing private source or target dimensions, or opening up new interaction pathways. We formalize these possibilities by decomposing each channel relative to the baseline AIS.

Let $P_{X}={\tilde{U}}_{A}{\tilde{U}}_{A}^{⊤}$ and $P_{Y}={\tilde{V}}_{A}{\tilde{V}}_{A}^{⊤}$ be orthogonal projectors onto the baseline source and target AIS, respectively, where ${\tilde{U}}_{A}$ and ${\tilde{V}}_{A}$ are orthonormal bases for $\text{col}\left(U_{A}\right)$ and $\text{col}\left(V_{A}\right)$. Using I=P+(I−P) on both the source and target sides, we decompose each channel’s rank-1 perturbation $\Delta A_{i}=w_{X,i}w_{Y,i}^{⊤}$ into four orthogonal components (see Appendix A.7):

(10) $$\Delta A_{i}=\underset{\Delta A_{i}^{ss}}{\underset{︸}{P_{X}\Delta A_{i}P_{Y}}}+\underset{\Delta A_{i}^{ns}}{\underset{︸}{\left(I-P_{X}\right)\Delta A_{i}P_{Y}}}+\underset{\Delta A_{i}^{sn}}{\underset{︸}{P_{X}\Delta A_{i}\left(I-P_{Y}\right)}}+\underset{\Delta A_{i}^{nn}}{\underset{︸}{\left(I-P_{X}\right)\Delta A_{i}\left(I-P_{Y}\right)}},$$

where superscripts denote same (s) or new (n) dimensions relative to the AIS in the source/target respectively, and the score $w_{Z,i}^{⊤}z$ is suppressed (it scales all components equally). Each component has a distinct interpretation (Fig.1c). $\Delta A_{i}^{ss}$: modulation reshapes interactions within the baseline AIS. $\Delta A_{i}^{ns}$: modulation makes source patterns outside the baseline AIS interact with target patterns within it. $\Delta A_{i}^{sn}$: modulation makes target patterns outside the baseline AIS interact with source patterns within it. $\Delta A_{i}^{nn}$: modulation creates new interaction pathways, outside the baseline AIS.

The four components are mutually orthogonal under the Frobenius inner product (Appendix A.7), yielding an additive decomposition of the channel strength ${\left\|\Delta A_{i}\right\|}_{F}^{2}$. We define fractional *geometry indices* as the fraction of channel strength carried by each component, $\alpha_{k}^{\left(i\right)}={\left\|\Delta A_{i}^{k}\right\|}_{F}^{2}\;/\;{\left\|\Delta A_{i}\right\|}_{F}^{2}$ for k∈{ss,ns,sn,nn}, thus having that $\sum_{k}\alpha_{k}^{\left(i\right)}=1$. Importantly, the indices depend only on the orientation of $w_{X,i}$ and $w_{Y,i}$ relative to the baseline subspaces, not on the modulator, and factorize into independent source and target alignment terms - e.g., $\alpha_{ss}^{\left(i\right)}=\cos^{2}\;\theta_{X}^{\left(i\right)}\cdot \cos^{2}\;\theta_{Y}^{\left(i\right)}$, where $\theta_{X}^{\left(i\right)}(\theta_{Y}^{\left(i\right)})$ is the angle between $w_{X,i}\left(w_{Y,i}\right)$. and the source (target) AIS - so that all four indices are determined by these two parameters alone (Appendix A.7.2). As a complement, we quantify each component’s contribution to ${\text{EV}}_{\text{mod}}$ by fitting constrained MICs that restrict modulation to lie within or outside the baseline subspaces. This reduces to the unconstrained problem on the AIS-projected $X^{\prime }$ and $Y^{\prime \prime }$ (Appendix A.8), enabling direct cross-validated comparison of ${\text{EV}}_{\text{mod}}$ across geometric components.

## 3 Validating MICs on simulated data

We validated our pipeline on simulated data, assessing MICs recovery, robustness to limited samples, and performance against simpler baselines. All details are reported in Appendix A.9.

### 3.1 Rank recovery, sample-efficiency, and benchmarks

**Figure 2:**
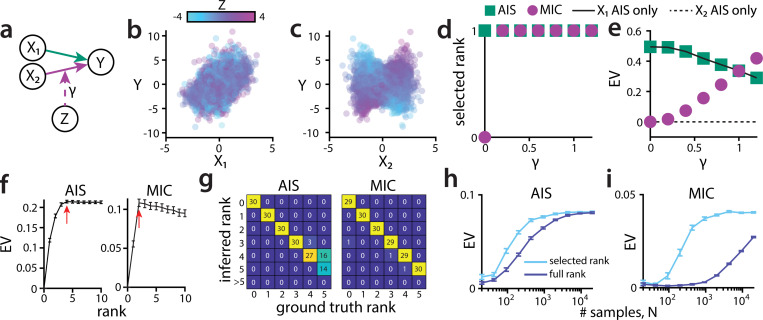
Testing MICs on simulated data. (a) Simulated scenario: a 2D source $X=\left(X_{1},X_{2}\right)$ interacts with a 1D target Y; the $X_{1}\to Y$ interaction is additive, whereas the $X_{2}\to Y$ interaction is multiplied by γZ, where γ is the parameter setting the modulation strength. (b) Joint distribution of $X_{1}$ and Y; dots color indicates the value of Z. (c) Same as (b) for $X_{2}$ and Y. (d) Selected AIS and modulation ranks as function of γ. (e) Explained Variance (EV) by the additive (green) and multiplicative (purple) channels vs γ. Solid and dashed black lines denote the AIS EV from $X_{1}$ and $X_{2}$ alone, respectively. (f) Model rank selection. AIS (left) and modulation (right) EV as function of the fitted rank (ground-truth ranks: $m_{A}=4$, left; $m_{C}=2$, right). Lines: mean ± SEM across cross-validation folds; red arrows: selected rank. (g) Heatmaps of ground-truth versus inferred ranks for the additive (left) and multiplicative (right) channels; 5 simulations per $\left(m_{A},m_{C}\right)$ ground-truth rank combination (30 per marginal rank). (h) Variance explained by AIS as a function of sample size N, for the rank-selected low-rank model (blue) and the full-rank model (cyan); 30 simulations per scenario, $n_{X}=n_{Y}=n_{Z}=10$; $m_{A}=2$, $m_{C}=1$. (i) Same as (h) for MICs.

To test whether MICs correctly quantify changes in source-target interactions as a function of a modulator Z, we first simulated a minimal scenario (Fig. 2a): a 2D source $X=\left(X_{1},X_{2}\right)$, a 1D target Y, and a 1D modulator Z. $X_{1}$ drove Y additively, independently of Z (Fig. 2b), while the $X_{2}\to Y$ interaction was gated by Z with strength γ (Fig. 2c). AIS correctly recovered a rank-1 subspace from X to Y, and MICs recovered a rank-1 modulation whenever γ>0 (Fig. 2d). MIC EV increased monotonically with γ, while AIS EV decreased as a growing fraction of the variance of Y was absorbed by the Z-gated interaction - confirming that AIS is blind to modulatory contributions (Fig. 2e). Fitting AIS on $X_{1}$ or $X_{2}$ alone made this explicit: AIS EV from $X_{1}$ matched the full- X AIS EV, whereas AIS EV from $X_{2}$ remained at zero regardless of γ (Fig. 2e, dashed lines).

Next, we considered high-dimensional sources, targets, and modulators $\left(n_{X}=n_{Y}=n_{Z}=10\right)$. We varied the ground-truth AIS and MIC ranks from 0 to 5 (5 simulations per rank combination), drawing $U_{A}$, $V_{A}$, $W_{X}$, $W_{Y}$, $W_{Z}$ at random for each simulation. For each fit, we selected the simplest model within 1 SEM of the maximum cross-validated EV (Fig. 2f, [16, 43]). Because this rule occasionally assigns positive AIS or MIC rank under $m_{A}=m_{C}=0$ ground truth (Fig. S1), we used a shuffled-residual permutation test to attribute positive ranks only when EV exceeded the p=0.05 threshold (2-fold CV; Appendix A.10). This procedure recovered AIS and MIC ground-truth ranks in most simulations (Fig. 2g). The dominant residual error was a mild under-estimation of AIS rank at the highest swept value $\left(m_{A}=5\right)$, suggesting that the pipeline selects AIS ranks conservatively. To tease apart the contributions of Step 1 and the significance test, we swept the $2^{4}=16$ combinations of presence or absence of the four model terms $m_{A}$, $m_{B}$, $m_{C}$, $m_{D}\in \{0,1\}$; Fig. S1a). The full pipeline recovered ground-truth $m_{A}$ and $m_{A}$ with high fidelity (4% and 0% false positives, respectively (Fig. S1b-c). Skipping Step 1 inflated $m_{A}$ whenever Z drove both X and Y, particularly when via the same Z patterns (Fig. S1d-e). Skipping the significance test inflated both $m_{A}$ and $m_{C}$ when the respective ground-truth ranks were zero (18% and 34% false positives; Fig. S1f-g). Skipping both was strictly worse than either alone (Fig. S1h-i).

We then assessed how AIS and MIC EV recovery scaled with sample size N and dimensionality $d=n_{X}=n_{Y}=n_{Z}$, against full-rank counterparts (no tensor-rank constraint on AIS or MICs). Reduced-rank fits saturated the ground-truth EV with substantially fewer samples in both cases (Fig. 2h-i and S2a-c, f-h). Normalized recovery curves collapsed onto N/d for both reduced-rank models (Fig. S2d, i), versus $N/d^{2}$ for full-rank AIS and $N/d^{3}$ for full-rank MIC (Fig. S2e, j), consistent with 𝒪(d) per-rank parameter counts against $𝒪\left(d^{2}\right)$ and $𝒪\left(d^{3}\right)$ for unconstrained AIS and MICs (Appendix A.2). Rank restriction therefore reduces the sample requirement by a factor up to d for AIS and $d^{2}$ for MIC. Moreover, MICs also tolerated misspecification of the residual noise model: replacing Gaussian emissions on X and Y with Poisson distribution often used to describe spike counts [44] - or more extreme zero-inflated Poisson preserved ${\mathrm{EV}}_{\text{mod}}$ scaling with overall modulation strength and recovery of ground truth MICs direction (Fig. S3).

Finally, since prior work examined how AIS changes across discrete levels of a third variable [3, 19, 45, 46], we benchmarked MICs versus a discretization approach. We partitioned Z into N quantile bins, refitted AIS within each bin, and computed the ${\mathrm{EV}}_{\text{mod}}$ as the gap between these binned fits and a single global AIS fit (“Z-split”; same CV folds). For 1D Z, ${\mathrm{EV}}_{\text{mod}}$ was nonmonotonic in N- rising as finer bins resolved Z-dependent structure, then collapsing as per-bin fits became data-starved - but its peak stayed below MIC ${\mathrm{EV}}_{\text{mod}}$ (Fig. S4a). For dim (Z)>1, where no natural discretization exists, Z-split applied to the leading PC of Z degraded sharply as additional Z dimensions misaligned with that PC, while MIC ${\mathrm{EV}}_{\text{mod}}$ remained stable (Fig. S4b).

Together, these results show that MICs efficiently and reliably recover low-dimensional modulation structure in high-dimensional settings - a component of Y to which AIS is blind.

### 3.2 Numerical validation of the geometric decomposition

**Figure 3:**
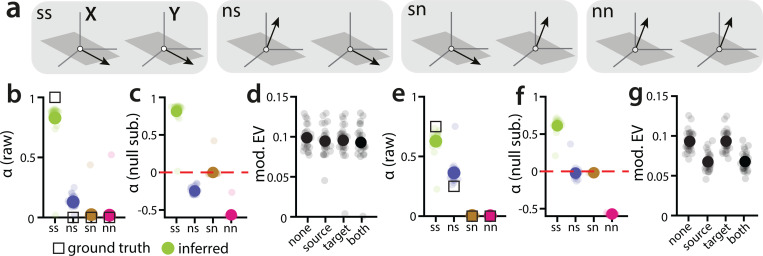
Validating the geometric decomposition on simulated MICs. (a) Schematics of the four geometric components (ss,ns,sn,nn) classifying how $w_{X,i}$ and $w_{Y,i}$ align with the baseline AIS (grey plane) on the source and target sides. (b) Raw geometric indices for a scenario with ground-truth $\alpha_{ss}=1$ and all others zero. Squares: ground truth; filled circles: mean across simulations; light dots: individual simulations. (c) Same data after subtracting the per-simulation random-rotation null (dashed line: α equal to expected value under the null). (d) Constrained-fit ${\mathrm{EV}}_{\text{mod}}$ with the indicated MIC weights constrained to lie in the baseline AIS:“source”: $w_{X}$; “target”: $w_{Y}$; “both”: both; “none”: unconstrained fit. (e-g) Same as (b-d) for a scenario with $\alpha_{ss}^{\text{GT}}=3/4$, $\alpha_{ns}^{\text{GT}}=1/4$. We ran 30 simulations in each scenario with 1000 samples each. $n_{X}=50$, $n_{Y}=5$, $n_{Z}=1$ in all simulations.

We next tested whether the geometry indices α and the constrained-fit ${\mathrm{EV}}_{\text{mod}}$ recover the correct MIC geometry with respect to AIS (Fig. 3a). All simulations used $n_{X}=50$, $n_{Y}=5$, and $n_{Z}=1$, implementing a source-target dimensional imbalance which may be present in brain recordings.

We first simulated a MIC entirely confined to the baseline AIS ($\alpha_{ss}^{\text{GT}}=1$, all others zero). Raw indices returned a large $\alpha_{ss}$ but also a systematic $\alpha_{ns}>0$ (Fig. 3b). This positive bias is a geometric artifact of the large difference between $n_{X}$ and the baseline source rank $m_{A}$: because $n_{X}-m_{A}$ dimensions are available orthogonal to the AIS, small fit-driven deviations of $w_{X,i}$ out of the baseline subspace inflate $\alpha_{ns}$. To correct for this, for each fitted MIC we drew $w_{X,i}$ and $w_{Y,i}$ independently and uniformly on their respective unit spheres, we recomputed the four indices, and subtracted the random-rotation null means from the raw α to obtain null-subtracted $\tilde{\alpha }$ (see Appendix A.7.3). After null subtraction only ${\tilde{\alpha }}_{ss}$ remained positive (Fig. 3c), consistent with ground truth. The constrained-fit ${\mathrm{EV}}_{\text{mod}}$ agreed: restricting the MIC to the ss component alone (both source and target MICs constrained to the respective AIS) recovered essentially all of the unconstrained ${\mathrm{EV}}_{\text{mod}}$ (Fig. 3d). We next considered a mixed geometry ($\alpha_{ss}^{\text{GT}}=3/4$ and $\alpha_{ns}^{\text{GT}}=1/4$). Raw indices correctly individuated both components (Fig. 3e), but null subtraction pushed ${\tilde{\alpha }}_{ns}$ around zero (Fig. 3f): the true ns contribution was on the order of the random alignment the null was calibrated to remove, so the test dismissed a genuine component. Constrained EV resolved the ambiguity: the ss-only fit recovered ≈3/4 of the unconstrained ${\mathrm{EV}}_{\text{mod}}$. The gap was closed by allowing MICs to lie outside AIS in the source (target constraint; $\alpha_{ns}>0$), but not by including sn (source constraint; Fig. 3g). A sweep over multi-MIC scenarios $\left(m_{C}>1\right)$ with randomly distributed ground-truth α further confirmed that geometry indices recover the correct geometry in more complex settings (Fig. S5).

Null-subtracted $\tilde{\alpha }$ and constrained-fit ${\mathrm{EV}}_{\text{mod}}$ are thus complementary: the former is closed-form and cheap, an efficient first-pass diagnostic that can provide false negatives; the latter is more expensive but directly and reliably quantifies each geometric term’s contribution to ${\mathrm{EV}}_{\text{mod}}$.

## 4 Behavioral state modulates prefrontal-visual cortical top-down interactions

**Figure 4:**
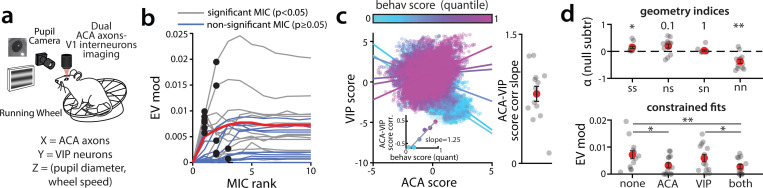
Application of MICs to real data. (a) Simultaneous imaging of ACA axons and VIP neurons in V1 with pupil and wheel measures. (b) Modulatory EV versus modulation rank; gray (blue) lines: individual sessions (n=28) with significant (non-significant) ${\mathrm{EV}}_{\text{mod}}$; red line: across-sessions mean; black dots: single-session selected rank. (c) Left: example MIC. Scatter of ACA versus VIP population activity, each projected onto the MIC (scores); dot color encodes quantiles of behavioral state score. Inset: Pearson correlation between ACA and VIP scores within each behavioral state score quantile. Right: average correlation slope across MICs; gray dots: sessions with significant ${\mathrm{EV}}_{\text{mod}}\;(n=13)$. (d) Top: null-subtracted geometry indices. Bottom: modulatory EV under different geometry constraints (ACA: source-in-AIS; VIP: target-in-AIS; both: both constraints). P-values show Bonferroni-corrected two-tailed one-sample t-tests in (d) top and repeated-measures ANOVA in (d) bottom. *, *p* < 0.05; **, *p* < 0.01. We plot mean ± SEM across sessions.

We validated MICs on recordings of real brain populations. Top-down projections from prefrontal cortex to primary visual cortex (V1) gate visual processing in a state-dependent manner [47–49]. Anterior cingulate (ACA, part of prefrontal cortex) axons have been shown to activate Vasoactive Intestinal Peptide (VIP) interneurons in V1 to modulate visual responses [47], and both ACA axonal activity and VIP neurons are strongly influenced by arousal and locomotion [49, 50]. However, whether and how behavioral state reshapes the prefrontal populations that interact top-down with visual cortical target populations is unknown. We thus asked whether behavioral state could modulate the interaction between specific activity patterns of ACA axons and VIP neurons in V1. We collected a new dataset of simultaneous two-photon imaging of ACA axonal projections in V1 and V1 VIP interneurons in head-fixed mice during passive visual stimulation (drifting gratings or natural movies), while tracking pupil diameter and running speed (Fig. 4a; Appendix A.11). We took ACA axons as source X and VIP neurons as target Y (following anatomical directionality), and defined the modulator $Z\in {\mathbb{R}}^{2}$ (hereafter *behavioral state*) as the joint vector of two well-established markers of behavioral state , pupil diameter and running speed [48, 51].

MICs identified significant ${\mathrm{EV}}_{\text{mod}}$ in 13 of 28 sessions (residual circular shuffle test on held-out sequential data halves; Fig. S6a-c; Appendix A.10). In these sessions, MICs accounted for (0.7±0.1)% (mean ± SEM) of total VIP population variance with mean selected rank $m_{C}=1.69$. Behavioral state thus modulates ACA-VIP interactions beyond driving each population alone (Fig. S6d-e), during both grating and natural-movie stimulation (Fig. S6f-g). The recovered MICs revealed that, consistently across sessions, the correlation between low-dimensional ACA-axon and VIP-neuron activity patterns increased with the projection of behavioral state onto $w_{Z,i}$ (Fig. 4c).

We next asked how the recovered MICs are organized relative to the baseline AIS. Decomposing each MIC into the four orthogonal geometric components (Eq. 10; Fig. 4d, top), we found that ${\tilde{\alpha }}_{ss}$ was significantly above zero (greater-than-chance alignment with the AIS in both populations), ${\tilde{\alpha }}_{ns}$ trended above zero (mild misalignment localized in ACA), and ${\tilde{\alpha }}_{nn}$ was significantly below zero (below-random engagement of patterns private to both populations). To assess whether geometric components within the random-alignment range contributed to modulation, we fitted geometry-constrained MICs (Fig. 4d, bottom). We found an asymmetric effect of behavioral state on ACA-VIP interactions. Constraining MICs to lie in the VIP AIS - which forces $\alpha_{sn}=\alpha_{nn}=0$- did not significantly reduce ${\mathrm{EV}}_{\text{mod}}$ relative to the unconstrained model, indicating that target-side deviations from the VIP AIS do not contribute to modulation. Conversely, constraining MICs to lie in the ACA AIS -which forces $\alpha_{ns}=\alpha_{nn}=0$- significantly reduced ${\mathrm{EV}}_{\text{mod}}$, and the loss was no larger when additionally enforcing $\alpha_{sn}=0$ (both-AIS constraint, leaving only $\alpha_{ss}$). These comparisons identify $\alpha_{ns}$ as the only geometric component beyond $\alpha_{ss}$ that contributes to behavioral-state modulation. Further analyses confirmed that the MIC-AIS alignment was significantly larger in VIP than in ACA (Fig. S6h), with MIC weights aligning strongly with the VIP AIS but weakly with the ACA AIS (Fig. S6j-k). Neither the better alignment in VIP nor the null-subtracted geometry indices were explained by the imbalance in population sizes between ACA axons and VIP interneurons (Fig. S6l-m). Both ${\mathrm{EV}}_{\text{mod}}$ and the recovered MIC geometry were robust to pipeline random initialization (Fig. S7).

Together, these analyses reveal that behavioral state predominantly reconfigures the ACA-axon patterns engaged in the interaction, while the coupled VIP patterns remain comparatively stable.

## 5 Discussion

We developed and validated MICs, a new method that extends the communication-subspace framework [16] to quantify how a multivariate modulator Z reshapes interactions between a source X and a target Y. The method models the effective source-target operator as the baseline AIS plus a Z-dependent perturbation, parametrized as a sum of rank-1 channels across source, target, and modulator. The low-rank structure makes the method tractable in the under-sampled, high-dimensional regime typical of modern neural data [8, 9]. Beside deriving MICs, we provide a cross-validated hierarchical fitting pipeline (Algorithm 1), a residual-shuffle significance test, and a closed-form decomposition of each MIC into four orthogonal geometric components relative to the baseline AIS. Together with the constrained-fit modulatory EV, these tools enable reliable detection and geometric characterization of modulation - whether it reshapes baseline interactions, recruits private dimensions of one population, or opens new interactions.

We validated MICs on both simulated and real neural data. On simulated data, the pipeline reliably recovered ground-truth modulation ranks and MICs geometry with respect to AIS, outperformed simpler approaches, and resolved modulatory EV with up to $d^{2}$ fewer samples than full-rank tensor regression. On real data, MICs revealed that behavioral state reconfigures the interactions between ACA-axon patterns and a stable set of V1 VIP patterns - beyond marginal modulation of either population alone. This asymmetry could reflect a top-down control architecture in which state-dependent recruitment of distinct ACA projection patterns is routed through a conserved VIP interface [47], eliciting a consistent disinhibitory local influence onto V1 pyramidal cells [52].

Several limitations point to natural extensions. MICs capture linear effects of Z on the source-target mapping. Extensions to nonlinear settings could be pursued along complementary routes. Generative latent variable models offer a data-intensive path, capturing nonlinear local dynamics while retaining linear inter-region channels [53]. Information-theoretic approaches based on partial information decomposition could quantify modulation through synergistic interactions sensitive to the full joint distribution [54, 55], though scaling to large multivariate populations remains a major challenge. Although our simulations indicate robustness to departures from Gaussianity, validation on electrophysiological recordings with low-rate spike counts - possibly via generalized CP frameworks [56] - remains an important next step. Moreover, similarly to RRR communication subspaces, MICs are intrinsically *correlational* [17] as they identify statistical dependencies at zero lag. Introducing MICs in time-lagged variants of communication subspace pipelines [57], combined with Wiener-Granger [11, 12], would allow MICs to quantify modulation of directional cross-area communication that could be better interpreted in *causal* terms. Furthermore, we currently take the baseline as the session-wide mean by working with zero-centered variables. Alternative schemes (e.g., anchoring the baseline at biologically interpretable values of the modulator) may improve biological interpretability.

By providing a compact, data-efficient, and theoretically grounded characterization of how a possibly high-dimensional modulator reshapes interactions between high-dimensional populations, MICs are well positioned to enable new discoveries about context-dependent interactions in neural systems.

## Supplementary Material

Supplement 1

## Data Availability

The analysis code and data supporting the findings of this study will be made publicly available upon acceptance. Researchers interested in early access can contact the lead contact.

## Footnotes

1 Unlike the RRR subspace - identifiable only as a column space - the CP components of the resulting tensor C satisfying Kruskal’s condition [41] are individually identifiable (Appendix A.3); hence we call them channels, not subspaces.
