# Supplementary material for: Decomposing the modulation of interactions between neuronal populations
Source: bioRxiv. 2026 May 18:2026.05.14.725145. Preprint. [Version 1] doi: 10.64898/2026.05.14.725145 (PMC13228498; doi:10.64898/2026.05.14.725145)
Supplement: Supplement 1 [file NIHPP2026.05.14.725145v1-supplement-1.pdf]

bioRxiv preprint doi: https://doi.org/10.64898/2026.05.14.725145; this version posted May 18, 2026. The copyright holder for this preprint (which was not certified by peer review) is the author/funder, who has granted bioRxiv a license to display the preprint in perpetuity. It is made available under a CC-BY 4.0 International license.

A Technical appendices

Throughout the appendix, we use the notation established in the main text: $\mathbf{X} \in \mathbb{R}^{N \times n_X}$ (source), $\mathbf{Y} \in \mathbb{R}^{N \times n_Y}$ (target), $\mathbf{Z} \in \mathbb{R}^{N \times n_Z}$ (modulator), all zero-centered column-wise. We use $\|\cdot\|_F$ for the Frobenius norm, $\langle K, L \rangle_F = \mathrm{tr}(K^\top L)$ for the Frobenius inner product, $\odot$ for the element-wise (Hadamard) product, and $\otimes$ for the outer (tensor) product.

A.1 Introduction to reduced-rank regression

We briefly summarize the ridge-regularized reduced-rank regression (ridge-RRR) estimator used throughout this work. For full derivations, see [40, 17].

Consider the linear model $\mathbf{Y} = \mathbf{X}A + \mathbf{E}$, where $A \in \mathbb{R}^{n_X \times n_Y}$ with $\mathrm{rank}(A) \le m$. We seek to minimize the ridge-regularized loss subject to a rank constraint:

$$\mathcal{L}_{\mathrm{RRR}}(A) = \|\mathbf{Y} - \mathbf{X}A\|_F^2 + \lambda \|A\|_F^2, \qquad \text{subject to } \mathrm{rank}(A) \le m. \tag{S1}$$

Full-rank ridge estimate. Ignoring the rank constraint, setting $\nabla_A \mathcal{L} = 0$ yields:

$$A_{\mathrm{ridge}} = (\mathbf{X}^\top \mathbf{X} + \lambda I)^{-1} \mathbf{X}^\top \mathbf{Y}. \tag{S2}$$

Rank-constrained projection. The key result (see [17], Appendices C-D, for a complete proof) is that incorporating the rank constraint is well approximated by projecting the ridge estimate onto the subspace spanned by the top m eigenvectors of a specific matrix. Concretely, the ridge-RRR solution is:

$$\hat{A} = A_{\mathrm{ridge}} V_r V_r^\top, \tag{S3}$$

where $V_r \in \mathbb{R}^{n_Y \times m}$ contains the top m eigenvectors of the symmetric positive semidefinite matrix $A_{\mathrm{ridge}}^\top \mathbf{X}^\top \mathbf{X} A_{\mathrm{ridge}}$. Equivalently, V_r is obtained by performing PCA on the $N \times n_Y$ matrix of ridge-regularized predictions $\mathbf{X} A_{\mathrm{ridge}}$ and retaining the top m components.

Interpretation: source and target subspaces. The solution in Eq. S3 has the factorized form $\hat{A} = U_A V_A^\top$, with:

$$V_A = V_r \in \mathbb{R}^{n_Y \times m}, \qquad U_A = A_{\mathrm{ridge}} V_r \in \mathbb{R}^{n_X \times m}. \tag{S4}$$

These factors have direct interpretations in terms of the relationship between source and target variables:

- **Source AIS**, $\mathrm{col}(U_A) \subset \mathbb{R}^{n_X}$: the m-dimensional subspace of source patterns that co-vary with the target. Source patterns orthogonal to this subspace are "private" - they have no linear relationship with the target.
- **Target AIS**, $\mathrm{col}(V_A) \subset \mathbb{R}^{n_Y}$: the m-dimensional subspace of target patterns that can be linearly related to the source. Target patterns orthogonal to this subspace are "private" and unexplained by the source.

Note that while the subspaces $\mathrm{col}(U_A)$ and $\mathrm{col}(V_A)$ are uniquely determined by $\hat{A}$, the individual columns of U_A and V_A are not: for any invertible $L \in \mathbb{R}^{m \times m}$, the alternative factorization $\hat{A} = (U_A L)(V_A L^{-\top})^\top$ yields the same product. This rotational ambiguity means that individual "communication dimensions" within the subspace are not identifiable from the model alone.

bioRxiv preprint doi: https://doi.org/10.64898/2026.05.14.725145; this version posted May 18, 2026. The copyright holder for this preprint (which was not certified by peer review) is the author/funder, who has granted bioRxiv a license to display the preprint in perpetuity. It is made available under a CC-BY 4.0 International license.

A.2 From scalar interaction regression to the MICs modulation tensor

In this section, we position the MICs model within the broader landscape of interaction modeling: we start from scalar regression with an interaction term, generalize to the high-dimensional setting where the interaction is encoded by a three-way tensor $\mathcal{C}$, recover MICs as a CP-rank constraint on $\mathcal{C}$, and finally relate it to prior tensor regression. To build intuition, we briefly connect our model to the familiar setting of regression with interaction terms. Consider first a scalar source $x \in \mathbb{R}$, scalar modulator $z \in \mathbb{R}$, and scalar target $y \in \mathbb{R}$. A standard interaction model is:

$$y = \beta_x x + \beta_z z + \beta_{xz}\, xz + \varepsilon, \tag{S5}$$

where $\beta_{xz}\, xz$ is the interaction term: the effect of x on y depends linearly on z, and vice versa.

Our model generalizes this to the high-dimensional setting where $x \in \mathbb{R}^{n_X}$, $y \in \mathbb{R}^{n_Y}$, $z \in \mathbb{R}^{n_Z}$. Without any dimensionality reduction, the target for observation n would be:

$$y_n = A^\top x_n + B^\top z_n + \sum_{j=1}^{n_X} \sum_{l=1}^{n_Z} (x_n)_j\, (z_n)_l\, c_{jl} + \varepsilon_n, \tag{S6}$$

where $c_{jl} \in \mathbb{R}^{n_Y}$ is a vector of interaction coefficients for source dimension j and modulator dimension l. This full interaction model has $n_X \times n_Z \times n_Y$ free parameters in the interaction term alone, which is prohibitively large in typical neuroscience settings (e.g., $n_X = n_Y = n_Z = 50$ gives 125,000 interaction parameters).

Equivalently, collecting the vectors $c_{jl} \in \mathbb{R}^{n_Y}$ into a single three-way array, i.e. *the full-rank modulation tensor*, $\mathcal{C} \in \mathbb{R}^{n_Y \times n_X \times n_Z}$ with entries $\mathcal{C}_{i,j,l} = (c_{jl})_i$, the modulation term in Eq. S6 is the contraction of $\mathcal{C}$ against the source and modulator vectors of observation n:

$$\sum_{j=1}^{n_X} \sum_{l=1}^{n_Z} c_{jl}\, (x_n)_j\, (z_n)_l \;=\; \mathcal{C} \times_2 x_n \times_3 z_n \;\in\; \mathbb{R}^{n_Y}. \tag{S7}$$

Here $\times_k$ denotes the *mode-k vector product*, i.e., contraction of a tensor along its k-th index: for $T \in \mathbb{R}^{I_1 \times \cdots \times I_N}$ and $u \in \mathbb{R}^{I_k}$, $(T \times_k u)_{i_1,\ldots,i_{k-1},i_{k+1},\ldots,i_N} = \sum_{i_k=1}^{I_k} T_{i_1,\ldots,i_N}\, u_{i_k}$ [33]. Mode 2 (size n_X) is thus contracted with the source x_n and mode 3 (size n_Z) with the modulator z_n, leaving mode 1 (size n_Y) as the output. Equivalently, $\mathcal{C}$ is evaluated on the rank-1 predictor $x_n \otimes z_n \in \mathbb{R}^{n_X \times n_Z}$ formed by source and modulator at observation n.

The MICs model (Eq. 5) greatly reduces the number of estimated parameters by imposing a low-rank structure on $\mathcal{C}$. Each MIC i contributes a rank-1 interaction:

$$y_n = A^\top x_n + B^\top z_n + \sum_{i=1}^{m_C} w_{Y,i} (w_{X,i}^\top x_n)(w_{Z,i}^\top z_n) + \varepsilon_n. \tag{S8}$$

This is exactly Eq. S6 with the three-way array of interaction coefficients constrained to have the CP decomposition $c_{jl} = \sum_{i=1}^{m_C} (w_{X,i})_j (w_{Z,i})_l\, w_{Y,i}$, reducing the number of interaction parameters from $n_X \times n_Z \times n_Y$ to $m_C(n_X + n_Z + n_Y)$. For $m_C = 3$ and $n_X = n_Y = n_Z = 50$, this is a compression from 125,000 to 450 parameters. Importantly, the low-rank structure is not merely a computational convenience: it provides interpretability. Each channel defines a specific source axis ($w_{X,i}$), modulator axis ($w_{Z,i}$), and target axis ($w_{Y,i}$), making it possible to ask which specific source patterns have their relationship with which specific target patterns modulated by which specific modulator patterns. In the scalar case (Eq. S5), there is only one such "channel" ($m_C = 1$, with $w_X = w_Z = w_Y = 1$, and the channel strength equals β_{xz}).

In isolation, the modulation term is a specific instance of the tensor-on-tensor regression framework of [38], with per-observation predictor tensor $x_n \otimes z_n \in \mathbb{R}^{n_X \times n_Z}$ and outcome $y_n \in \mathbb{R}^{n_Y}$. The rank-1 structure of the predictor - the outer product of two distinct observed variables rather than a single multiway array - is what endows the CP decomposition with its bilinear-interaction

bioRxiv preprint doi: https://doi.org/10.64898/2026.05.14.725145; this version posted May 18, 2026. The copyright holder for this preprint (which was not certified by peer review) is the author/funder, who has granted bioRxiv a license to display the preprint in perpetuity. It is made available under a CC-BY 4.0 International license.

interpretation. The full MICs model (Eq. 5) embeds this term inside an additive RRR framework with residualized source $X' = X - ZD$, which falls outside [38]: Lock's framework fits a single coefficient tensor governing one predictor, and does not address the disentangling of additive from multiplicative effects of distinct predictors X and Z. Beyond the model itself, the hierarchical pipeline (Algorithm 1) handles the simultaneous presence of additive and multiplicative effects of Z under shared cross-validation folds, and the geometric decomposition (Section 2.4) exploits the existence of a baseline AIS operator A against which each MIC is projected - neither of which has a counterpart in [38].

A.3 Uniqueness of MICs: Kruskal's theorem

The use of *channel* rather than *subspace* to describe MICs reflects an algebraic distinction from AIS. Reduced-rank matrix factorizations identify column subspaces but not individual factor columns, which carry rotational indeterminacy. The three-way tensor structure of $\mathcal{C}$ breaks this indeterminacy: under a condition on the Kruskal ranks of the factor matrices, the individual rank-1 components of the CP decomposition are essentially unique, and identifiable up to permutation and per-channel rescaling (Kruskal's theorem). We state the theorem, verify the condition in our setting, and note its practical consequences for MIC analysis.

A.3.1 Kruskal rank and theorem statement

Definition A.1. The *Kruskal rank* of a matrix $M \in \mathbb{R}^{n\times r}$, denoted $k(M)$, is the largest integer j such that every subset of j columns of M is linearly independent.

Note that $k(M) \leq \text{rank}(M) \leq \min(n, r)$, with equality $k(M) = \text{rank}(M)$ when every subset of rank(M) columns is linearly independent. For a matrix whose columns are drawn independently from a continuous distribution on $\mathbb{R}^n$ with $n \geq r$, $k(M) = r$ with probability one.

Theorem A.2 (Kruskal, 1977 [41]). *Let $\mathcal{C} = \sum_{i=1}^{m_C} w_{Y,i} \otimes w_{X,i} \otimes w_{Z,i}$ be a rank-m_C CP decomposition with factor matrices $W_Y \in \mathbb{R}^{n_Y \times m_C}$, $W_X \in \mathbb{R}^{n_X \times m_C}$, $W_Z \in \mathbb{R}^{n_Z \times m_C}$. If*

$$k(W_Y) + k(W_X) + k(W_Z) \geq 2m_C + 2, \tag{S9}$$

then the decomposition is essentially unique*: any other rank-m_C decomposition is related to it by a permutation of the channels and per-channel rescaling.*

In settings where, $n_X, n_Y, n_Z \gg m_C$ and the weight vectors are estimated from data with continuous noise, generically $k(W_Y) = k(W_X) = k(W_Z) = m_C$. Eq. S9 then reduces to $3m_C \geq 2m_C + 2$, i.e., $m_C \geq 2$ (uniqueness for $m_C = 1$ is trivial up to an overall scalar). This argument breaks when any factor dimension is small relative to m_C. For example, $k(W_Z) = 1$ for a scalar modulator ($n_Z = 1$), so Eq. S9 fails for all $m_C \geq 2$.

A.3.2 Practical implications for MIC analysis

Many natural analyses with AIS are subspace-level - for example, predicting the target response evoked by a given source pattern - and do not require identifiability of individual factor columns. Kruskal's theorem provides a stronger guarantee that becomes relevant when multiple MICs with distinct geometries coexist in the same population. Without column-level identifiability, two channels with different geometric profiles (e.g., one predominantly α_{ss}, another predominantly α_{ns}) could be rotated into mixtures, blurring their distinct alignment with the baseline AIS. Theorem A.2 ensures that this cannot happen: each channel retains its individual geometry. We do not fully exploit this property in the present manuscript - our current analyses summarize geometric structure at the level of MIC-averaged indices - but it opens the door to per-channel

bioRxiv preprint doi: https://doi.org/10.64898/2026.05.14.725145; this version posted May 18, 2026. The copyright holder for this preprint (which was not certified by peer review) is the author/funder, who has granted bioRxiv a license to display the preprint in perpetuity. It is made available under a CC-BY 4.0 International license.

comparative analyses (e.g., contrasting the biological correlates of channels with different α profiles) that we view as a promising direction for future work.

The CP parametrization has an inherent per-channel scaling ambiguity: $(w_{X,i}, w_{Z,i}, w_{Y,i})$ and $(w_{X,i}/\beta,\ \beta\gamma\, w_{Z,i},\ w_{Y,i}/\gamma)$ define the same channel, since the outer product is multilinear. Under the identifiability conditions of Theorem A.2, this is the *only* remaining ambiguity. We resolve this by extracting unit-norm directions and absorbing the magnitude into a single *channel strength* $\sigma_i = \|w_{X,i}\| \cdot \|w_{Z,i}\| \cdot \|w_{Y,i}\|$, giving $\mathcal{C} = \sum_i \sigma_i\, \bar{w}_{Y,i} \otimes \bar{w}_{X,i} \otimes \bar{w}_{Z,i}$. This normalization is used throughout the paper when displaying or comparing MIC weights and scores (e.g., Fig. 4c, Fig. S6j-k). It does not affect the geometry indices α (which depend only on directions) or the channel's contribution to $\mathrm{EV}_{\mathrm{mod}}$.

A.4 The CP Frobenius penalty

The modulation tensor $\mathcal{C} \in \mathbb{R}^{n_Y \times n_X \times n_Z}$, with CP decomposition $\mathcal{C} = \sum_{i=1}^{m_C} w_{Y,i} \otimes w_{X,i} \otimes w_{Z,i}$, has squared Frobenius norm:

$$\begin{aligned}
\|\mathcal{C}\|_F^2 &= \sum_{j,k,l} \mathcal{C}_{jkl}^2 = \sum_{j,k,l} \left(\sum_{i=1}^{m_C} (w_{Y,i})_j\, (w_{X,i})_k\, (w_{Z,i})_l \right)^2 \\
&= \sum_{j,k,l} \sum_{i=1}^{m_C} \sum_{r=1}^{m_C} (w_{Y,i})_j (w_{Y,r})_j \cdot (w_{X,i})_k (w_{X,r})_k \cdot (w_{Z,i})_l (w_{Z,r})_l \\
&= \sum_{i,r} (w_{Y,i}^\top w_{Y,r})(w_{X,i}^\top w_{X,r})(w_{Z,i}^\top w_{Z,r}) \\
&= \sum_{i,r} (G_Y)_{ir}\, (G_X)_{ir}\, (G_Z)_{ir} \\
&= \langle G_Y,\, G_X \odot G_Z \rangle_F = \mathrm{tr}\Big(G_Y^\top (G_X \odot G_Z) \Big)\,, \qquad \text{(S10)}
\end{aligned}$$

where $G_Y = W_Y^\top W_Y$, $G_X = W_X^\top W_X$, $G_Z = W_Z^\top W_Z \in \mathbb{R}^{m_C \times m_C}$ are the Gram matrices of the factor matrices, and the last line uses the identity $\sum_{ir} A_{ir} B_{ir} C_{ir} = \langle A, B \odot C \rangle_F$ for the element-wise product. This expression is efficient to compute (it involves only $m_C \times m_C$ matrices) and is used in the regularization term of the modulation loss (Eq. 8 in the main text).

A.5 ALS derivation for the modulation term

In this section, we derive ALS for the modulation loss of our model (Eq. 8). ALS for ridge-regularized CP-rank tensor regression is an established tool [33, 37, 38]. The closed-form updates derived below follow from specializing this framework - specifically the non-separable L_2 penalty $\lambda_C \|\mathcal{C}\|_F^2$ on the CP coefficient tensor of [38] - to the bilinear structure of Eq. 8. We provide the derivation for self-containedness and summarize it in algorithm 2.

The modulation loss of our model with the penalty term expressed as in Eq. A.4 is:

$$\mathcal{L}(W_X, W_Z, W_Y) = \|\mathbf{Y}'' - \Phi\, W_Y^\top\|_F^2 + \lambda_C\, \mathrm{tr}\Big(G_Y^\top (G_X \odot G_Z) \Big)\,, \qquad \text{(S11)}$$

where $\Phi = (\mathbf{X}' W_X) \odot (\mathbf{Z} W_Z) \in \mathbb{R}^{N \times m_C}$, and $\mathbf{Y}'' = \mathbf{Y}' - \mathbf{X}' A$ are the residuals from the additive model. Each ALS sweep updates W_Y, then W_X, then W_Z, while holding the other two fixed, similarly to the ALS procedures for CP-rank-constrained tensor regression developed in [37, 38].

Expanding the first terms in the RHS of Eq.S11 yields:

$$\|\mathbf{Y}'' - \Phi W_Y^\top\|_F^2 = \mathrm{tr}(\mathbf{Y}''^\top \mathbf{Y}'') - 2\,\mathrm{tr}(W_Y \Phi^\top \mathbf{Y}'') + \mathrm{tr}(W_Y \Phi^\top \Phi W_Y^\top), \qquad \text{(S12)}$$

and, in the following, we refer to the two terms that depend on the factor matrices as the cross-term $-2\,\mathrm{tr}(W_Y \Phi^\top \mathbf{Y}'')$ and the quadratic term $\mathrm{tr}(W_Y \Phi^\top \Phi W_Y^\top)$.

bioRxiv preprint doi: https://doi.org/10.64898/2026.05.14.725145; this version posted May 18, 2026. The copyright holder for this preprint (which was not certified by peer review) is the author/funder, who has granted bioRxiv a license to display the preprint in perpetuity. It is made available under a CC-BY 4.0 International license.

A.5.1 Update for W_Y

With W_X and W_Z (and hence Φ, G_X, G_Z) fixed, the loss is quadratic in W_Y. Taking the derivative of Eq.S11 with respect to $W_Y \in \mathbb{R}^{n_Y \times m_C}$ and setting it to zero yields:

$$\frac{\partial \mathcal{L}}{\partial W_Y} = -2\,\mathbf{Y}''^{\top}\Phi + 2\,W_Y\Phi^{\top}\Phi + 2\lambda_C\,W_Y(G_X \odot G_Z). \tag{S13}$$

The derivative of the penalty term (i.e. the second term in the RHS of Eq.S11) follows from $\frac{\partial}{\partial W_Y}\mathrm{tr}(W_Y^{\top}W_Y(G_X \odot G_Z)) = 2\,W_Y(G_X \odot G_Z)$, since $G_X \odot G_Z$ is symmetric. Setting the $\frac{\partial \mathcal{L}}{\partial W_Y}$ derivative to zero gives:

$$\boxed{W_Y^{\top} = \left(\Phi^{\top}\Phi + \lambda_C\,(G_X \odot G_Z)\right)^{-1}\Phi^{\top}\mathbf{Y}''.} \tag{S14}$$

This is an $m_C \times m_C$ linear system (one right-hand side per target dimension), which is inexpensive to solve.

A.5.2 Update for W_X

With W_Z and W_Y fixed, the columns of W_X are coupled through both the Hadamard product in Φ and the CP penalty. We derive the gradient via total differentials.

Define $K_Z = \mathbf{Z}W_Z \in \mathbb{R}^{N \times m_C}$ and $K_Y = \mathbf{Y}''W_Y \in \mathbb{R}^{N \times m_C}$. Considering a perturbation $W_X \to W_X + dW_X$, we compute the three contributions to $d\mathcal{L}$ coming from the cross-term, the quadratic term and the penalty term respectively:

$$d\mathcal{L} = d\mathcal{L}_{\text{cross}} + d\mathcal{L}_{\text{quad}} + d\mathcal{L}_{\text{pen}}. \tag{S15}$$

(i) Cross term $-2\,\mathrm{tr}(W_Y\Phi^{\top}\mathbf{Y}'')$: Under $d\Phi = (\mathbf{X}'\,dW_X) \odot K_Z$ and using the Hadamard product identity $\langle A, B \odot C\rangle_F = \langle A \odot B, C\rangle_F$:

$$-2\,\mathrm{tr}(W_Y\,d\Phi^{\top}\mathbf{Y}'') = -2\,\mathrm{tr}\Big((K_Y \odot K_Z)^{\top}\mathbf{X}'\,dW_X\Big). \tag{S16}$$

(ii) Quadratic term $\mathrm{tr}(G_Y\Phi^{\top}\Phi)$:

$$2\,\mathrm{tr}(G_Y\,\Phi^{\top}d\Phi) = 2\,\mathrm{tr}\Big((\Phi G_Y \odot K_Z)^{\top}\mathbf{X}'\,dW_X\Big). \tag{S17}$$

(iii) Penalty term: Since $dG_X = dW_X^{\top}W_X + W_X^{\top}dW_X$,

$$\lambda_C\,d\Big[\mathrm{tr}(G_Y^{\top}(G_X \odot G_Z))\Big] = 2\lambda_C\,\mathrm{tr}\Big([W_X(G_Y \odot G_Z)]^{\top}\,dW_X\Big). \tag{S18}$$

Therefore,

$$d\mathcal{L} = -2\,\mathrm{tr}\Big((K_Y \odot K_Z)^{\top}\mathbf{X}'\,dW_X\Big) + 2\,\mathrm{tr}\Big((\Phi G_Y \odot K_Z)^{\top}\mathbf{X}'\,dW_X\Big) + 2\lambda_C\,\mathrm{tr}\Big([W_X(G_Y \odot G_Z)]^{\top}\,dW_X\Big). \tag{S19}$$

Combining and identifying the gradient of the scalar loss via $d\mathcal{L} = \mathrm{tr}(\nabla_{W_X}\mathcal{L}^{\top}\,dW_X)$:

$$\frac{\partial \mathcal{L}}{\partial W_X} = 2\,\mathbf{X}'^{\top}[(\Phi G_Y - K_Y) \odot K_Z] + 2\lambda_C\,W_X(G_Y \odot G_Z). \tag{S20}$$

bioRxiv preprint doi: https://doi.org/10.64898/2026.05.14.725145; this version posted May 18, 2026. The copyright holder for this preprint (which was not certified by peer review) is the author/funder, who has granted bioRxiv a license to display the preprint in perpetuity. It is made available under a CC-BY 4.0 International license.

Block linear system. Setting Eq. S20 to zero does not yield a decoupled update for the columns of W_X, because the columns are coupled through ΦG_Y and through the penalty term. Let $L = G_Y \odot G_Z$ and $H = \mathbf{X}'^{\top}(K_Y \odot K_Z) \in \mathbb{R}^{n_X \times m_C}$. Writing $w_r = W_X^{(:,r)}$, $k_{Z,r} = K_Z^{(:,r)} \in \mathbb{R}^N$, and $h_j = H^{(:,j)}$, the stationarity condition for the j-th column is

$$\sum_{r=1}^{m_C} \Big[(G_Y)_{rj}\, \mathbf{X}'^{\top} \mathrm{diag}(k_{Z,r} \odot k_{Z,j})\, \mathbf{X}' + \lambda_C\, L_{rj}\, I_{n_X}\Big]\, w_r = h_j. \tag{S21}$$

where I_{n_X} denotes the $n_X \times n_X$ identity matrix. Stacking the columns of W_X yields the linear system

$$\boxed{\mathbf{M}_X \,\mathrm{vec}(W_X) = \mathrm{vec}(H),} \tag{S22}$$

where $\mathbf{M}_X \in \mathbb{R}^{n_X m_C \times n_X m_C}$ is a symmetric block matrix with (j,r)-th block ($n_X \times n_X$ each)

$$\mathbf{M}_X^{(jr)} = (G_Y)_{rj}\, \mathbf{X}'^{\top} \mathrm{diag}(k_{Z,r} \odot k_{Z,j})\, \mathbf{X}' + \lambda_C\, L_{rj}\, I_{n_X}. \tag{S23}$$

The diagonal matrix need not be formed explicitly: for any $v \in \mathbb{R}^N$,

$$\mathbf{X}'^{\top} \mathrm{diag}(v)\, \mathbf{X}' = \mathbf{X}'^{\top} \big(\mathrm{diag}(v)\mathbf{X}'\big),$$

i.e. by scaling the rows of $\mathbf{X}'$ by v before left-multiplying by $\mathbf{X}'^{\top}$.

A.5.3 Update for W_Z

By symmetry between $\mathbf{X}'$ and $\mathbf{Z}$ in the Hadamard product $\Phi = (\mathbf{X}'W_X) \odot (\mathbf{Z}W_Z)$, the update for W_Z is obtained by swapping $(\mathbf{X}', W_X, G_X)$ with $(\mathbf{Z}, W_Z, G_Z)$. Defining $K_X = \mathbf{X}'W_X$ (using the just-updated W_X), $L' = G_Y \odot G_X$, and $H' = \mathbf{Z}^{\top}(K_Y \odot K_X)$:

$$\boxed{\mathbf{M}_Z \,\mathrm{vec}(W_Z) = \mathrm{vec}(H'),} \tag{S24}$$

with (j,r)-th block

$$\mathbf{M}_Z^{(jr)} = (G_Y)_{rj}\, \mathbf{Z}^{\top} \mathrm{diag}(k_{X,r} \odot k_{X,j})\, \mathbf{Z} + \lambda_C\, L'_{rj}\, I_{n_Z},$$

where $k_{X,r} = K_X^{(:,r)}$.

It is worth noticing that the ALS objective in Eq. S11 is non-convex. In principle, different initializations may converge to different local optima. Empirically, on the ACA-VIP data both $\mathrm{EV}_{\mathrm{mod}}$ and the recovered MIC geometry were stable across random ALS initializations (Fig. S7; Appendix A.11).

A.6 Hierarchical model fitting algorithm

For clarity, we summarize the full fitting pipeline in Algorithm 1. The algorithm mirrors the structure of the implementation described in the main text and in the code: first fit the additive effects of the modulator on source and target, then fit the baseline AIS on the residualized variables, and finally fit the MICs on the residuals of the baseline model.

In the pseudocode below, calligraphic symbols denote collections of fold-specific quantities across cross-validation splits. For example, $\mathcal{B} = \{B^{(k)}\}_{k=1}^K$ denotes the collection of $Z \to Y$ regression matrices obtained from each fold k, and $\mathcal{Z} = \{(Z_{\mathrm{train}}^{(k)}, Z_{\mathrm{test}}^{(k)})\}_{k=1}^K$ denotes the collection of train/test splits of Z. Algebraic expressions involving calligraphic variables indicate fold-wise operations. For instance,

$$\mathcal{Z}\mathcal{B} = \{(Z_{\mathrm{train}}^{(k)} B^{(k)},\ Z_{\mathrm{test}}^{(k)} B^{(k)})\}_{k=1}^K. \tag{S25}$$

bioRxiv preprint doi: https://doi.org/10.64898/2026.05.14.725145; this version posted May 18, 2026. The copyright holder for this preprint (which was not certified by peer review) is the author/funder, who has granted bioRxiv a license to display the preprint in perpetuity. It is made available under a CC-BY 4.0 International license.

Algorithm 1 Hierarchical fitting procedure for MICs

```
function FullFit(X, Y, Z)
    Split data into K cross-validation folds:
        {(X_train^(k), X_test^(k), Y_train^(k), Y_test^(k), Z_train^(k), Z_test^(k))}_{k=1}^K
    for k = 1, ..., K do
        z-score the k-th train and test sets using the mean and standard deviation of the k-th training set
    end for
    (B, m_B) ← RRRFit(Z, Y)
    (D, m_D) ← RRRFit(Z, X)
    X' ← X − Z D
    Y' ← Y − Z B
    (A, m_A) ← RRRFit(X', Y')
    Y'' ← Y' − X' A
    (W_X, W_Z, W_Y, m_C) ← CFit(X', Y'', Z)
    return (A, B, W_X, W_Z, W_Y, D, m_A, m_B, m_C, m_D)
end function

function RRRFit(X, Y)
    for m = 1, ..., min(n_X, n_Y) do
        λ* ← ridge penalty λ that maximizes average fold-wise EV of rank-m X → Y regression
            matrix A(m, λ) (MATLAB bayesopt)
        EV(m) ← across-fold EV for λ = λ*
        sem(m) ← across-fold standard error of mean EV for λ = λ*
        A(m) ← A(m, λ*)
    end for
    m* ← smallest m such that EV(m) + sem(m_max) ≥ EV(m_max), where m_max is the rank of
            max EV
    return (A(m*), m*)
end function

function CFit(X', Y'', Z)
    for m = 1, ..., max rank do
        λ* ← Frobenius penalty λ that maximizes average fold-wise EV of rank-m MICs
        (W_X(m, λ), W_Z(m, λ), W_Y(m, λ)) obtained using Algorithm 2 (MATLAB bayesopt)
        EV(m) ← across-fold EV for λ = λ*
        sem(m) ← across-fold standard error of mean EV for λ = λ*
        (W_X(m), W_Z(m), W_Y(m)) ← (W_X(m, λ*), W_Z(m, λ*), W_Y(m, λ*))
    end for
    m* ← smallest m such that EV(m) + sem(m_max) ≥ EV(m_max), where m_max is the rank of
            max EV
    return (W_X(m*), W_Z(m*), W_Y(m*), m*)
end function
```

Algorithm 1 gives the global structure of the fitting pipeline. The final step, CFit, requires solving the low-rank tensor regression problem for the modulation term. We summarize the complete ALS procedure used inside CFit in Algorithm 2. The weight matrices are initialized

bioRxiv preprint doi: https://doi.org/10.64898/2026.05.14.725145; this version posted May 18, 2026. The copyright holder for this preprint (which was not certified by peer review) is the author/funder, who has granted bioRxiv a license to display the preprint in perpetuity. It is made available under a CC-BY 4.0 International license.

randomly (from a standard normal distribution), and sweeps are repeated until the relative change in the loss falls below a tolerance ϵ or a maximum number of sweeps $maxIter$ is reached (in our implementation we set $\epsilon = 10^{-5}$ and $maxIter = 100$).

Algorithm 2 ALS for fitting MICs

Require: Residuals $\mathbf{Y}'' \in \mathbb{R}^{N \times n_Y}$, residualized source $\mathbf{X}' \in \mathbb{R}^{N \times n_X}$, modulator $\mathbf{Z} \in \mathbb{R}^{N \times n_Z}$, rank m_C, penalty λ_C, tolerance ϵ, max sweeps T

Ensure: Weight matrices $W_X \in \mathbb{R}^{n_X \times m_C}$, $W_Z \in \mathbb{R}^{n_Z \times m_C}$, $W_Y \in \mathbb{R}^{n_Y \times m_C}$

Initialize W_X, W_Z, W_Y randomly
Compute $K_X \leftarrow \mathbf{X}' W_X,\ K_Z \leftarrow \mathbf{Z} W_Z,\ \Phi \leftarrow K_X \odot K_Z$
Compute Gram matrices $G_X \leftarrow W_X^\top W_X,\ G_Z \leftarrow W_Z^\top W_Z$
for $t = 1, \ldots, T$ **do**
  Update W_Y: $W_Y^\top \leftarrow \left(\Phi^\top \Phi + \lambda_C (G_X \odot G_Z)\right)^{-1} \Phi^\top \mathbf{Y}''$ ▷ Eq. S14
  Update $G_Y \leftarrow W_Y^\top W_Y,\ K_Y \leftarrow \mathbf{Y}'' W_Y$
  Update W_X: Assemble $\mathbf{M}_X$ and H; solve $\mathbf{M}_X \operatorname{vec}(W_X) = \operatorname{vec}(H)$ ▷ Eq. S22
  Update $K_X \leftarrow \mathbf{X}' W_X,\ \Phi \leftarrow K_X \odot K_Z,\ G_X \leftarrow W_X^\top W_X$
  Update W_Z: Assemble $\mathbf{M}_Z$ and H'; solve $\mathbf{M}_Z \operatorname{vec}(W_Z) = \operatorname{vec}(H')$ ▷ Eq. S24
  Update $K_Z \leftarrow \mathbf{Z} W_Z,\ \Phi \leftarrow K_X \odot K_Z,\ G_Z \leftarrow W_Z^\top W_Z$
  Compute loss $\mathcal{L}^{(t)}$ via Eq. S11
  if $|\mathcal{L}^{(t)} - \mathcal{L}^{(t-1)}| / |\mathcal{L}^{(t-1)}| < \epsilon$ **then**
    break
  end if
end for

The computational cost per sweep is dominated by assembling and solving the $n_X m_C \times n_X m_C$ and $n_Z m_C \times n_Z m_C$ block systems for W_X and W_Z: $\mathcal{O}((n_X m_C)^2 N + (n_X m_C)^3)$ and analogously for W_Z. The W_Y update costs only $\mathcal{O}(N m_C n_Y + m_C^3)$.

A.7 Geometric decomposition of MICs

In this section, we derive the four-component decomposition of each channel ΔA_i and the resulting form of the geometry indices:

$$\alpha_{ss}^{(i)} = \frac{\|\Delta A_i^{ss}\|_F^2}{\|\Delta A_i\|_F^2}, \qquad \alpha_{ns}^{(i)} = \frac{\|\Delta A_i^{ns}\|_F^2}{\|\Delta A_i\|_F^2}, \qquad \alpha_{sn}^{(i)} = \frac{\|\Delta A_i^{sn}\|_F^2}{\|\Delta A_i\|_F^2}, \qquad \alpha_{nn}^{(i)} = \frac{\|\Delta A_i^{nn}\|_F^2}{\|\Delta A_i\|_F^2}. \tag{S26}$$

The results in this section follow from standard algebra of orthogonal projectors (see, e.g., [58]) and are reported here for clarity and completeness; we do not claim them as novel theoretical contributions.

A.7.1 Orthogonal decomposition

The decomposition $M = M^{ss} + M^{ns} + M^{sn} + M^{nn}$ (Eq. 10) follows by inserting $I = P + (I - P)$ on both sides of M:

$$M = \left[P_X + (I - P_X)\right] M \left[P_Y + (I - P_Y)\right]. \tag{S27}$$

The pairwise orthogonality of the four components under the Frobenius inner product is a consequence of the idempotence of orthogonal projectors. For any two components that differ in their source-side projector (e.g., M^{ss} and M^{ns}):

$$\langle M^{ss}, M^{ns} \rangle_F = \operatorname{tr}\left(P_Y M^\top \underbrace{P_X (I - P_X)}_{=0} M P_Y \right) = 0, \tag{S28}$$

bioRxiv preprint doi: https://doi.org/10.64898/2026.05.14.725145; this version posted May 18, 2026. The copyright holder for this preprint (which was not certified by peer review) is the author/funder, who has granted bioRxiv a license to display the preprint in perpetuity. It is made available under a CC-BY 4.0 International license.

and for components that differ in their target-side projector (e.g., M^{ss} and M^{sn}), the analogous identity using $(I-P_Y)P_Y = 0$ and the cyclic property of the trace applies. The Pythagorean norm decomposition $\|M\|_F^2 = \|M^{ss}\|_F^2 + \|M^{ns}\|_F^2 + \|M^{sn}\|_F^2 + \|M^{nn}\|_F^2$ is an immediate consequence.

A.7.2 Explicit form of α indices for rank-1 perturbations

For a rank-1 matrix $M = w_X w_Y^\top$, the four components remain rank-1 (or zero):

$$M^{ss} = (P_X w_X)(P_Y w_Y)^\top, \quad M^{ns} = ((I - P_X)w_X)(P_Y w_Y)^\top, \quad \text{etc.,} \tag{S29}$$

since orthogonal projectors act linearly: $P_X(w_X w_Y^\top)P_Y = (P_X w_X)(P_Y w_Y)^\top$. Using the identity $\|ab^\top\|_F^2 = \|a\|^2\|b\|^2$ for rank-1 matrices (which follows from $\|ab^\top\|_F^2 = \text{tr}(ba^\top ab^\top) = (a^\top a)(b^\top b)$), the squared Frobenius norms factorize:

$$\|M^{ss}\|_F^2 = \|P_X w_X\|^2\|P_Y w_Y\|^2, \qquad \|M\|_F^2 = \|w_X\|^2\|w_Y\|^2, \tag{S30}$$

and analogously for the other components. The geometry indices (Eq. S26) therefore take the factorized form:

$$\alpha_{ss} = \cos^2\theta_X \cdot \cos^2\theta_Y, \qquad \alpha_{ns} = \sin^2\theta_X \cdot \cos^2\theta_Y, \tag{S31}$$

$$\alpha_{sn} = \cos^2\theta_X \cdot \sin^2\theta_Y, \qquad \alpha_{nn} = \sin^2\theta_X \cdot \sin^2\theta_Y, \tag{S32}$$

where $\cos^2\theta_X = \|P_X w_X\|^2/\|w_X\|^2$ is the squared cosine of the angle between w_X and the source AIS, and $\cos^2\theta_Y = \|P_Y w_Y\|^2/\|w_Y\|^2$ analogously for the target. The Pythagorean identity for projections ensures $\cos^2\theta + \sin^2\theta = 1$ on each side, so the four α values sum to one.

Remark A.3 (Independence from the modulator). Since each MIC i contributes $\Delta A_i(z) = (w_{Z,i}^\top z)\, w_{X,i} w_{Y,i}^\top$, the scalar $(w_{Z,i}^\top z)^2$ appears in both numerator and denominator of each α index and cancels. The geometry indices thus characterize a fixed geometric property of each channel, regardless of the instantaneous value of z or the modulator weight vector $w_{Z,i}$.

Remark A.4 (Two-parameter structure). The product form reveals that the full 2×2 table of α values is determined by just two free parameters: the source alignment $\cos^2\theta_X$ and the target alignment $\cos^2\theta_Y$. Defining $a_X = \cos^2\theta_X$ and $a_Y = \cos^2\theta_Y$:

$$\alpha_{ss} = a_X a_Y, \quad \alpha_{ns} = (1-a_X)a_Y, \quad \alpha_{sn} = a_X(1-a_Y), \quad \alpha_{nn} = (1-a_X)(1-a_Y). \tag{S33}$$

A.7.3 Null randomized test of α indices

Raw geometry indices α_k are subject to a systematic bias: finite-sample estimation causes the fitted directions $\mathbf{w}_{X,i}$, $\mathbf{w}_{Y,i}$ to deviate from their true orientations, and the dimensional structure of the problem ($n_X \gg m_A$ in typical recordings) determines where those deviations land - predominantly outside the AIS, inflating components such as α_{ns} and α_{nn} even when the true geometry is confined to α_{ss} (Fig. 3b).

To correct for this, we estimate the expected α values under a *random-direction null*: for each fitted MIC i, we replace $\mathbf{w}_{X,i}$ and $\mathbf{w}_{Y,i}$ with independent draws from the uniform distribution on their respective unit spheres, recompute the four indices, and repeat for N_{shuf} shuffles. The null-subtracted index is

$$\tilde{\alpha}_k^{(i)} = \alpha_k^{(i)} - \langle \alpha_k^{\text{null}} \rangle, \qquad k \in \{ss,\, ns,\, sn,\, nn\}, \tag{S34}$$

where $\langle\cdot\rangle$ denotes the mean over shuffles.

Values $\tilde{\alpha}_k > 0$ indicates that the fitted MIC aligns with geometric component k more than expected for a uniformly random direction in the same ambient space - that is, the channel exhibits geometric structure in that component beyond chance. The test is conservative: components whose true weight is small relative to the random-alignment baseline will be dismissed Fig. 3f), in which case constrained-fit EV_{mod} Appendix A.8) provides a more sensitive characterization of MICs geometry.

bioRxiv preprint doi: https://doi.org/10.64898/2026.05.14.725145; this version posted May 18, 2026. The copyright holder for this preprint (which was not certified by peer review) is the author/funder, who has granted bioRxiv a license to display the preprint in perpetuity. It is made available under a CC-BY 4.0 International license.

A.8 Constrained fitting: modulation within or outside baseline subspaces

As described in Section 3.2, we test whether modulatory variance is attributable to specific geometric components by fitting constrained models. The following result shows that constraining MIC factors to specified subspaces reduces, up to a constant, to an unconstrained problem on projected data, so that Algorithm 2 can be reused without modification.

A.8.1 General reduction

Proposition A.5 (Constrained fitting reduction)**.** *Let $Q_X \in \mathbb{R}^{n_X \times d_X}$ and $Q_Y \in \mathbb{R}^{n_Y \times d_Y}$ be semi-orthogonal matrices ($Q_X^\top Q_X = I_{d_X}$, $Q_Y^\top Q_Y = I_{d_Y}$), and consider the modulation loss (Eq. S11) constrained by the parametrization*

$$W_X = Q_X \widetilde{W}_X, \qquad W_Y = Q_Y \widetilde{W}_Y, \tag{S35}$$

with $\widetilde{W}_X \in \mathbb{R}^{d_X \times m_C}$ and $\widetilde{W}_Y \in \mathbb{R}^{d_Y \times m_C}$ free. Then the constrained loss is equivalent, up to an additive constant independent of the free parameters, to the unconstrained modulation loss applied to the projected data $(\mathbf{X}' Q_X,\ \mathbf{Y}'' Q_Y,\ \mathbf{Z})$.

Proof. Substituting Eq. S35 into the modulation loss and splitting the data-fit term using the fact that the prediction $\hat{\mathbf{Y}}$ lies entirely in $\mathrm{col}(Q_Y)$:

$$\|\mathbf{Y}'' - \hat{\mathbf{Y}}\|_F^2 = \|\mathbf{Y}'' Q_Y Q_Y^\top - \hat{\mathbf{Y}}\|_F^2 + \|\mathbf{Y}''(I - Q_Y Q_Y^\top)\|_F^2. \tag{S36}$$

The second term is constant in the free parameters. For the first, $Q_Y^\top Q_Y = I_{d_Y}$ gives $\|\mathbf{Y}'' Q_Y - [(\mathbf{X}' Q_X \widetilde{W}_X) \odot (\mathbf{Z} W_Z)] \widetilde{W}_Y^\top\|_F^2$. The penalty simplifies analogously: $Q_X^\top Q_X = I_{d_X}$ and $Q_Y^\top Q_Y = I_{d_Y}$ give Gram matrices $\widetilde{G}_X = \widetilde{W}_X^\top \widetilde{W}_X$ and $\widetilde{G}_Y = \widetilde{W}_Y^\top \widetilde{W}_Y$, so $\|\mathcal{C}\|_F^2 = \mathrm{tr}(\widetilde{G}_Y^\top(\widetilde{G}_X \odot G_Z))$. Combining:

$$\mathcal{L}_{\text{con.}}(\widetilde{W}_X, W_Z, \widetilde{W}_Y) = \left\| \mathbf{Y}'' Q_Y - \left[(\mathbf{X}' Q_X \widetilde{W}_X) \odot (\mathbf{Z} W_Z) \right] \widetilde{W}_Y^\top \right\|_F^2 + \lambda_C \|\widetilde{\mathcal{C}}\|_F^2, \tag{S37}$$

which is the unconstrained modulation loss in the projected variables. □ □

Eq. S37 can be minimized using the same ALS procedure (Algorithm 2). After optimization, the full-space factors are recovered via $W_X = Q_X \widetilde{W}_X$ and $W_Y = Q_Y \widetilde{W}_Y$.

A.8.2 Specific geometric constraints

The geometric components of the α decomposition correspond to specific choices of (Q_X, Q_Y). We list all eight possible constraints below for completeness. In the present paper we use three of these constraints, applied hierarchically to identify specific geometrical contributions to $\mathrm{EV}_{\mathrm{mod}}$ (Sections 2.4 and 4): the both-in-AIS (α_{ss}-only) fit, the source-in-AIS fit (w_X in the source AIS, w_Y free; forces $\alpha_{ns} = \alpha_{nn} = 0$), and the target-in-AIS fit (w_Y in the target AIS, w_X free; forces $\alpha_{sn} = \alpha_{nn} = 0$). Comparing the cross-validated $\mathrm{EV}_{\mathrm{mod}}$ across nested constrained and unconstrained fits attributes specific geometric components: for example, a significant gap between the both-in-AIS and source-in-AIS fits identifies a contribution from α_{sn}, a gap between the both-in-AIS and target-in-AIS fits identifies a contribution from α_{ns}, and a contribution to α_{nn} is implied when both the source-in-AIS→unconstrained and target-in-AIS→unconstrained gaps are significant. Fig. S5e-h illustrates this for the four individual ground-truth geometries: when $\alpha_{ss} = 1$ the both-in-AIS constraint captures the unconstrained $\mathrm{EV}_{\mathrm{mod}}$; when $\alpha_{ns} = 1$ ($\alpha_{sn} = 1$) only the target-in-AIS (source-in-AIS) constrain captures the unconstrained $\mathrm{EV}_{\mathrm{mod}}$, while constraining the source (target) reduces $\mathrm{EV}_{\mathrm{mod}}$ to zero; when $\alpha_{nn} = 1$ any constraint reduces $\mathrm{EV}_{\mathrm{mod}}$ to zero. The α attribution from this approach is not univocal - nested gaps quantify the variance explained by adding a geometric component on top of a constrained baseline,

bioRxiv preprint doi: https://doi.org/10.64898/2026.05.14.725145; this version posted May 18, 2026. The copyright holder for this preprint (which was not certified by peer review) is the author/funder, who has granted bioRxiv a license to display the preprint in perpetuity. It is made available under a CC-BY 4.0 International license.

rather than yielding a closed-form additive decomposition of $\mathrm{EV}_{\mathrm{mod}}$ into the four α terms - but comparison between these constrained models suffices to localize which components carry modulatory variance.

**Constraint**	Q_X	Q_Y
$\alpha_{ss} = 1$ (same source, same target)	$\tilde{U}_A$	$\tilde{V}_A$
$\alpha_{ns} = 1$ (new source, same target)	$\tilde{U}_A^\perp$	$\tilde{V}_A$
$\alpha_{sn} = 1$ (same source, new target)	$\tilde{U}_A$	$\tilde{V}_A^\perp$
$\alpha_{nn} = 1$ (new source, new target)	$\tilde{U}_A^\perp$	$\tilde{V}_A^\perp$
$\alpha_{nn} = \alpha_{sn} = 0$ (target in AIS)	—	$\tilde{V}_A$
$\alpha_{nn} = \alpha_{ns} = 0$ (source in AIS)	$\tilde{U}_A$	—
$\alpha_{ss} = \alpha_{ns} = 0$ (target out of AIS)	—	$\tilde{V}_A^\perp$
$\alpha_{ss} = \alpha_{sn} = 0$ (source out of AIS)	$\tilde{U}_A^\perp$	—

Here $\tilde{U}_A^\perp \in \mathbb{R}^{n_X \times (n_X - m_A)}$ and $\tilde{V}_A^\perp \in \mathbb{R}^{n_Y \times (n_Y - m_A)}$ are orthonormal bases for the orthogonal complements of the baseline source and target AIS, and "—" indicates that the corresponding factor is left unconstrained. Constraining to the "same" subspaces projects the data onto m_A-dimensional subspaces, making the ALS block systems much cheaper (block size drops from $n_X \times n_X$ to $m_A \times m_A$).

Remark A.6 (Constrained fitting vs. post-hoc α indices). The constrained model directly optimizes modulation within a specified geometric subspace, whereas the post-hoc α indices decompose an unconstrained solution. These can give different results: the unconstrained fit might place a channel at an intermediate angle (e.g., $\alpha_{ss} \approx \alpha_{ns} \approx 0.5$), whereas the constrained α_{ss} model finds the best purely within-subspace solution. Comparing the cross-validated $\mathrm{EV}_{\mathrm{mod}}$ of constrained versus unconstrained models provides a direct test of whether modulatory variance is attributable to specific geometric components, complementing the descriptive α indices.

A.9 Simulation details

In this section, we provide additional implementation details on all simulations of the paper. Simulations in Figs. 2, 3, S1, and S2, S3, S4, and S5 all share the generative structure

$$\begin{aligned}
\mathbf{Z} &= E_{\mathbf{Z}} \in \mathbb{R}^{N \times n_Z} \\
\mathbf{X} &= \mathbf{Z}D + E_{\mathbf{X}} \in \mathbb{R}^{N \times n_X} \\
\mathbf{Y} &= \mathbf{X}A + \mathbf{Z}B + \big[(\mathbf{X}W_X) \odot (\mathbf{Z}W_Z)\big] W_Y^\top + E_{\mathbf{Y}} \in \mathbb{R}^{N \times n_Y},
\end{aligned} \tag{S38}$$

but differ in interaction structure (A, B, D, W_X, W_Y, W_Z), population dimensionalities (n_X, n_Y, n_Z), and sample size N. Noise terms $E_{\mathbf{X}}, E_{\mathbf{Y}}, E_{\mathbf{Z}}$ are i.i.d. Gaussian with unit variance unless stated otherwise. The low-rank factors are parametrized as

$$A = U_A V_A^\top, \quad B = U_B V_B^\top, \quad D = U_D V_D^\top,$$

where the columns of $U_A \in \mathbb{R}^{n_X \times m_A}$, $V_A \in \mathbb{R}^{n_Y \times m_A}$, $U_B \in \mathbb{R}^{n_Z \times m_B}$, $V_B \in \mathbb{R}^{n_Y \times m_B}$, $U_D \in \mathbb{R}^{n_Z \times m_D}$, $V_D \in \mathbb{R}^{n_X \times m_D}$, and the MIC weight vectors $\mathbf{w}_X, \mathbf{w}_Y, \mathbf{w}_Z$ are drawn independently and uniformly from the unit sphere, unless the simulation specifies a particular geometry. Throughout, rank selection uses the 1-SEM rule applied to 10-fold cross-validated EV curves.

Modulation strength sweep; Fig. 2(a-e). We first considered a minimal scenario with $n_X = 2$, $n_Y = 1$, $n_Z = 1$, and $N = 10^4$. The AIS matrix is fixed at $A = [1\ 0]^\top$, so that only the first source dimension drives Y additively (independent of Z), while the second drives

bioRxiv preprint doi: https://doi.org/10.64898/2026.05.14.725145; this version posted May 18, 2026. The copyright holder for this preprint (which was not certified by peer review) is the author/funder, who has granted bioRxiv a license to display the preprint in perpetuity. It is made available under a CC-BY 4.0 International license.

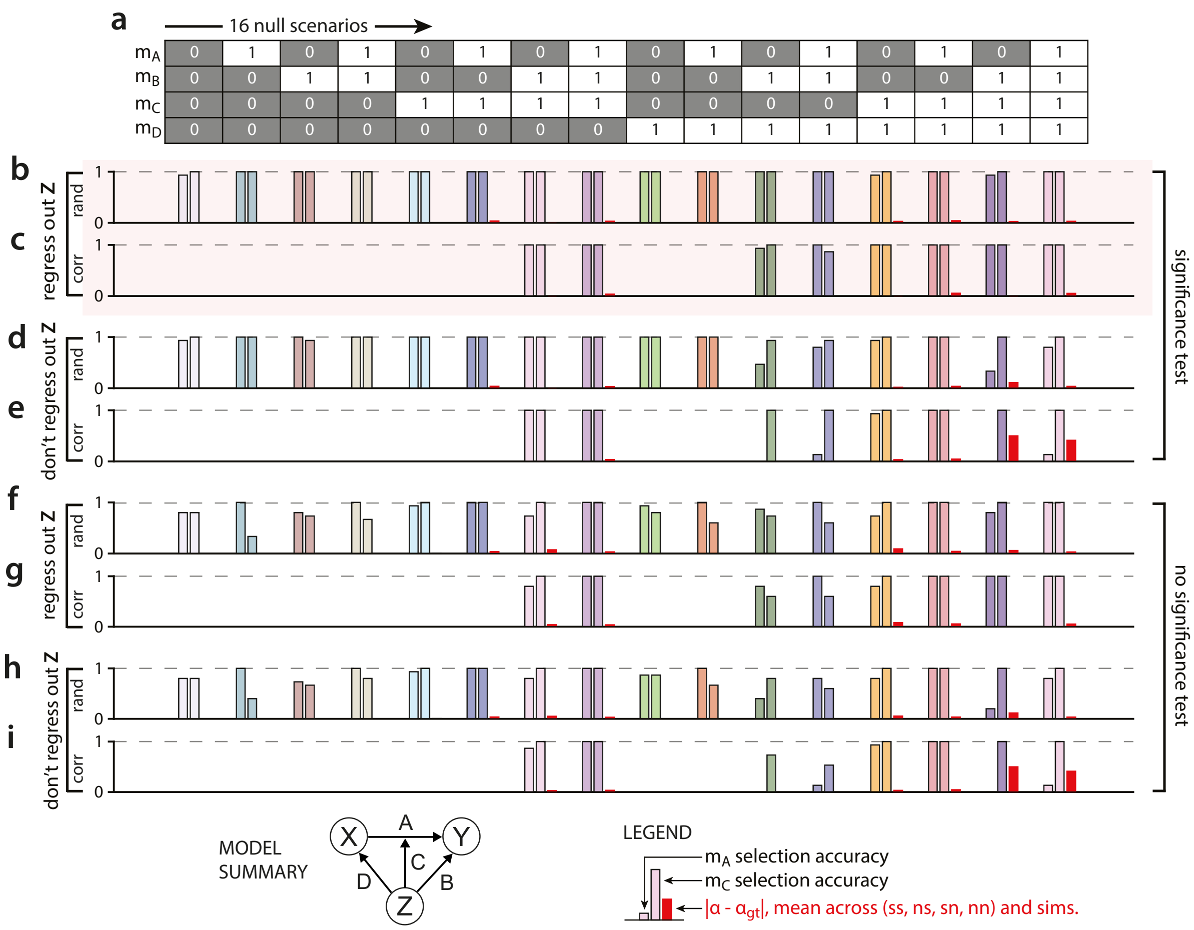

Figure S1: **Pipeline validation across all 16 rank combinations.** (a) Ground-truth rank indicator matrix: $m_A, m_B, m_C, m_D \in \{0, 1\}$ for each of the 16 simulated scenarios (columns). The model schematic (bottom left) is reproduced from Fig. 1b for reference. Panels (b-i) evaluate the MICs fitting pipeline across a 2×2×2 factorial design: whether Z is regressed out of X and Y (Step 1 of Algorithm 1; panels b-e vs. f-i), whether the shuffle significance test defined in App. A.10 is applied to EV_{mod} (panels b-c, f-g vs. d-e, h-i), and whether ground-truth interaction subspace vectors are drawn uniformly from the unit sphere (*rand*) or set to be perfectly co-aligned across model terms (*corr*; see below). In each sub-panel, bars for each scenario show m_A selection accuracy (left bar), m_C selection accuracy (center bar), and mean absolute geometry index error, averaged over the four geometric components (ss, ns, sn, nn) and simulations (right bar, red). (b) Pipeline with Step 1 and permutation significance test (*rand* condition): ground-truth weight vectors are drawn independently and uniformly from the unit sphere. (c) Same as (b), *corr* condition: for scenarios in which at least two of m_B, m_C, m_D are non-zero, the relevant low-rank components are set to be perfectly co-aligned. Specifically, when $m_B = m_C = 1$, the MIC and the $Z - Y$ interaction subspace are perfectly aligned in both Z and Y; when $m_D = m_C = 1$, the MIC and the $Z - X$ interaction subspace are perfectly aligned in both Z and X; when $m_B = m_D = 1$, the $Z \rightarrow Y$ and $Z \rightarrow X$ interaction subspaces are perfectly aligned in Z. (d-e) Same as (b-c) but without regressing Z out of X and Y (Step 1 omitted). (f-g) Same as (b-c) but without the permutation significance test. (h-i) Same as (b-c) but without Step 1 and without the permutation significance test. We ran 15 independent simulations per scenario, with $N = 2000$ samples per simulation and $n_X = n_Y = n_Z = 10$ in all simulations.

bioRxiv preprint doi: https://doi.org/10.64898/2026.05.14.725145; this version posted May 18, 2026. The copyright holder for this preprint (which was not certified by peer review) is the author/funder, who has granted bioRxiv a license to display the preprint in perpetuity. It is made available under a CC-BY 4.0 International license.

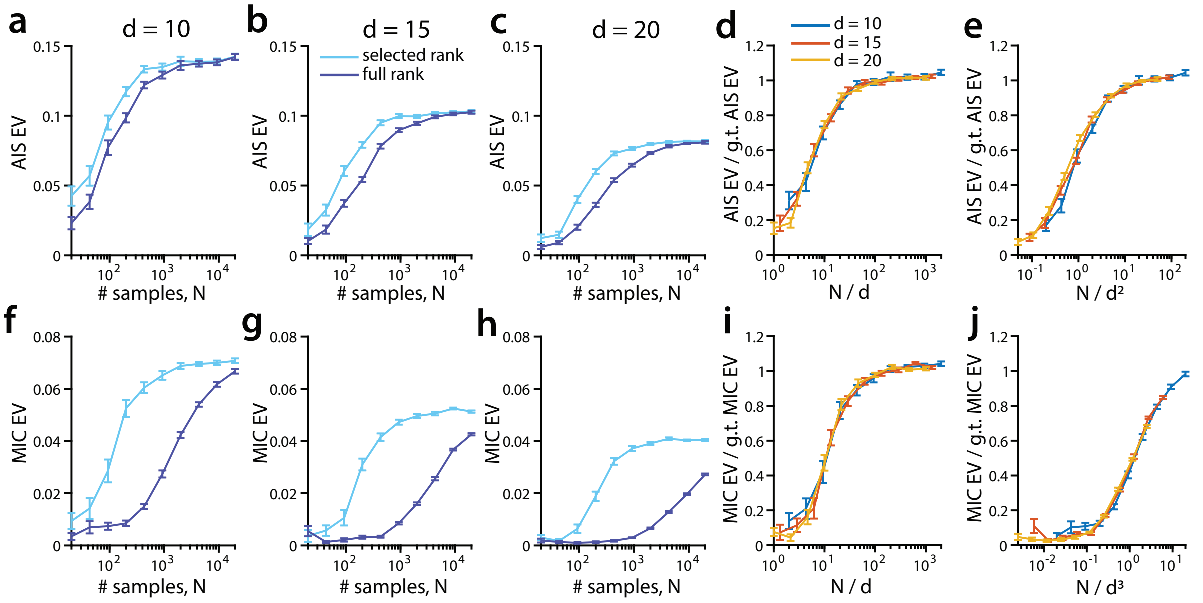

Figure S2: **Data efficiency of MICs relative to full-rank models.** (a) Variance explained by AIS as a function of sample size N, for the rank-selected low-rank model (blue) and the full-rank model (cyan), with $n_X = n_Y = n_Z = 10 =: d$. Lines show mean ± SEM across 30 independent simulations. (b-c) Same as (a), with $d = 15$ and $d = 20$. (d) Scaling collapse of low-rank AIS explained variance for the three values of d in (a-c). The number of samples is rescaled by a factor proportional to the number of parameters in the low-rank AIS model (specifically, $1/d$) and the explained variance is rescaled by the explained variance of the ground-truth AIS. (e) Same as (d), for the full-rank AIS model ($\propto d^2$ parameters). (f-h) Same as (a-c), for the low-rank and full rank MIC models. (i-j) Same as (d-e) for the low-rank (number of parameters $\propto d$) and full-rank (number of parameters $\propto d^3$) MIC models. Here the explained variance is rescaled by the explained variance of the ground-truth MIC. In all simulations, $m_A = 2$ and $m_C = 1$.

bioRxiv preprint doi: https://doi.org/10.64898/2026.05.14.725145; this version posted May 18, 2026. The copyright holder for this preprint (which was not certified by peer review) is the author/funder, who has granted bioRxiv a license to display the preprint in perpetuity. It is made available under a CC-BY 4.0 International license.

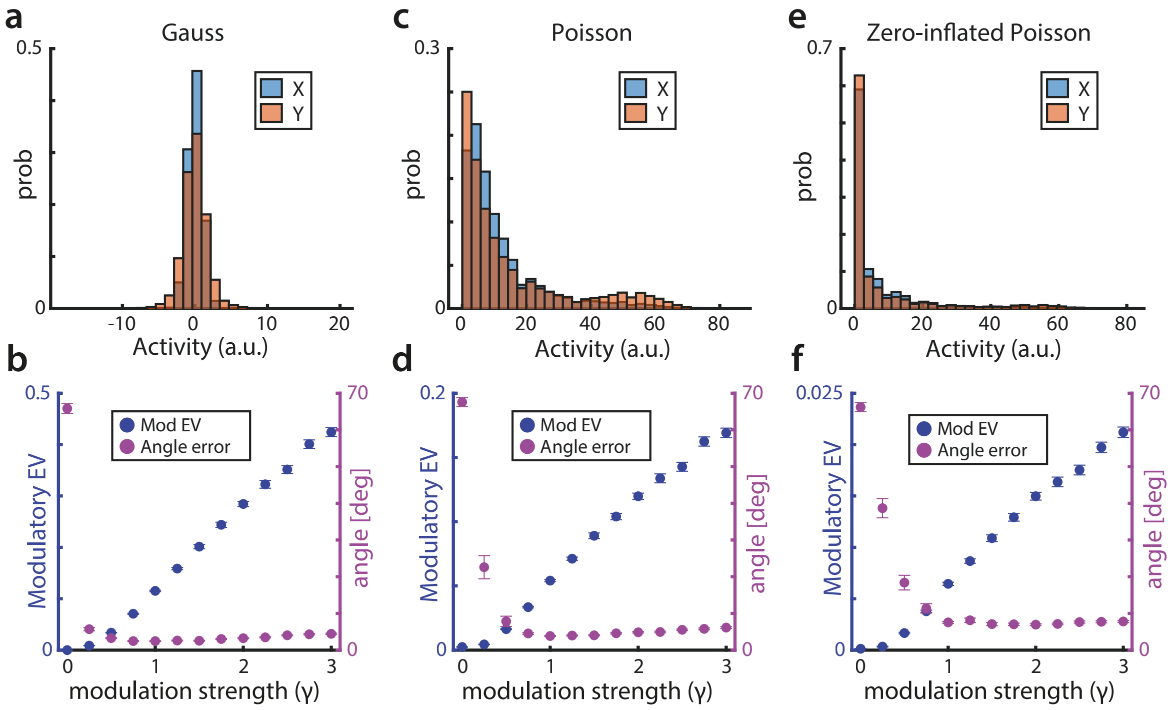

Figure S3: **MICs performance under non-Gaussian observation models.** All conditions share the same latent bilinear generative model, only the emission distribution of X and Y varies across scenarios. (a) Marginal distribution of X and Y for one example simulation in the Gaussian (linear benchmark) condition. (b) $\mathrm{EV}_{\mathrm{mod}}$ (blue) and mean angle between estimated and ground-truth MIC weight vectors (purple) as a function of modulation strength γ. (c-d) Same as (a-b) for Poisson-distributed X and Y. (e-f) Same as (a-b) for zero-inflated Poisson observations (zero-inflation probability $p = 0.5$). $n_X = n_Y = n_Z = 5$; ground-truth ranks $m_A = m_B = m_C = 1$, $m_D = 0$; $N = 10{,}000$ samples per simulation. Curves show mean $\pm$ SEM across 50 independent simulations.

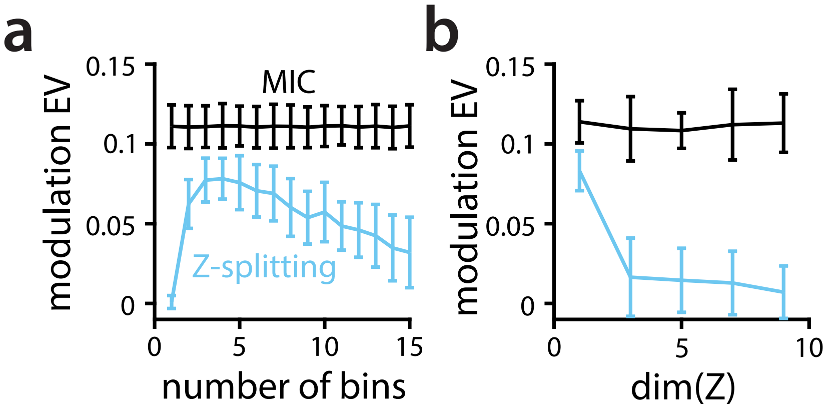

Figure S4: **Benchmarking MICs versus Z-splitting.** (a) Modulatory EV recovered by the MICs method (black) versus a benchmark that discretizes a 1D Z into quantile bins and refits the AIS within each bin (light blue), as a function of the number of bins. (b) Same comparison as a function of $\dim(Z)$, with the quantile-splitting benchmark applied to the first principal component of Z; 100 simulations per scenario, $N = 500$ samples per simulation.

bioRxiv preprint doi: https://doi.org/10.64898/2026.05.14.725145; this version posted May 18, 2026. The copyright holder for this preprint (which was not certified by peer review) is the author/funder, who has granted bioRxiv a license to display the preprint in perpetuity. It is made available under a CC-BY 4.0 International license.

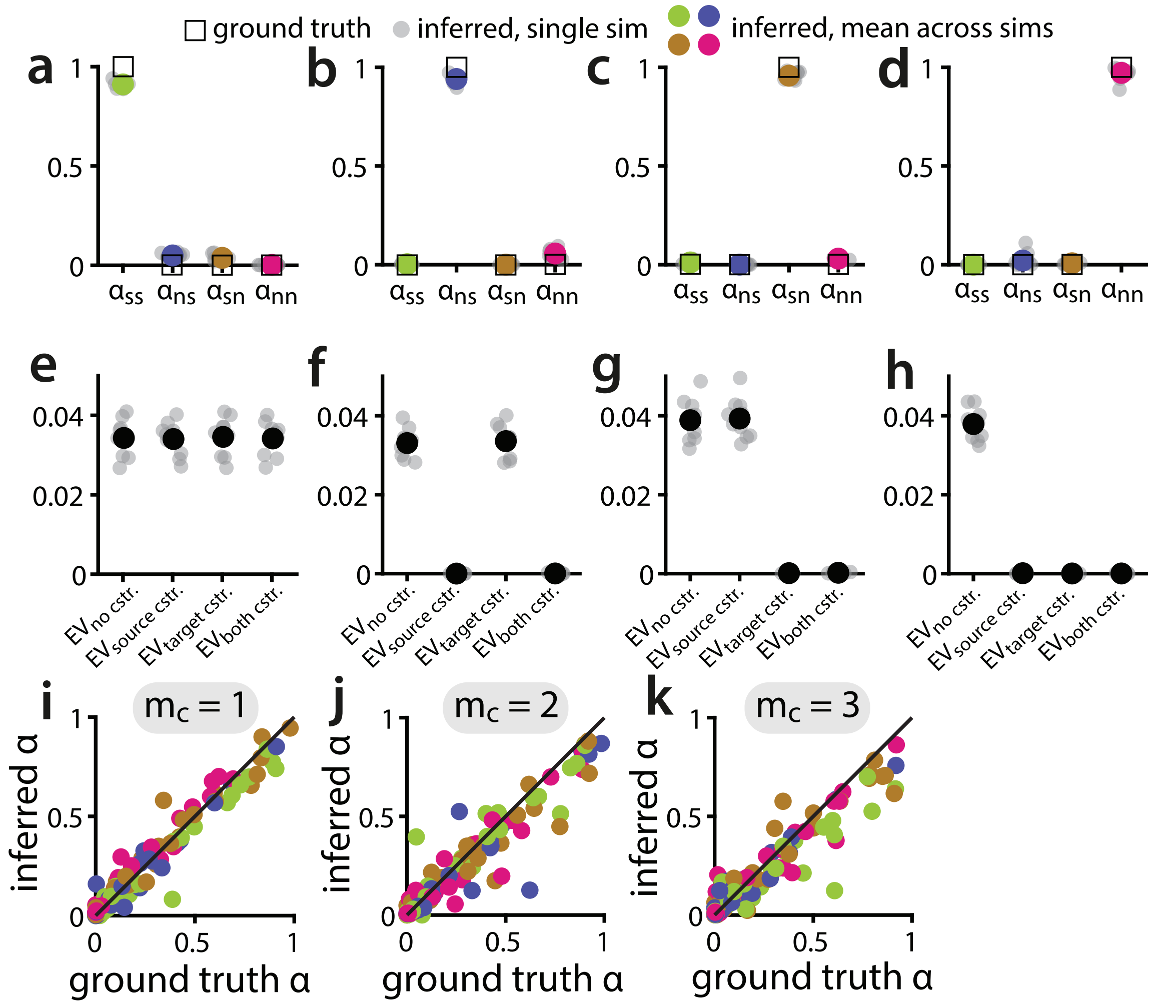

Figure S5: **Validating the geometric decomposition across single-component and multi-MIC scenarios.** (a) Raw geometry indices for a single-MIC scenario with ground-truth $\alpha_{ss}^{\mathrm{GT}} = 1$, all other components zero. Open squares: ground truth; filled colored circles: mean across simulations; light grey dots: individual simulations. (b-d) Same as (a) for ground-truth $\alpha_{ns}^{\mathrm{GT}} = 1$ (b), $\alpha_{sn}^{\mathrm{GT}} = 1$ (c), and $\alpha_{nn}^{\mathrm{GT}} = 1$ (d). (e) Constrained-fit $\mathrm{EV}_{\mathrm{mod}}$ for the $\alpha_{ss}^{\mathrm{GT}} = 1$ scenario, evaluated under four constraints: *no cstr.*, unconstrained fit; *source cstr.*, source factor confined AIS, forcing $\alpha_{ns} = \alpha_{nn} = 0$; *target cstr.*, target confined to AIS, forcing $\alpha_{sn} = \alpha_{nn} = 0$; *both cstr.*, both source and target confined to AIS, leaving only α_{ss} admissible. Filled black circles: mean across simulations; light grey dots: individual simulations. (f-h) Same as (e) for the $\alpha_{ns}^{\mathrm{GT}} = 1$ (f), $\alpha_{sn}^{\mathrm{GT}} = 1$ (g), and $\alpha_{nn}^{\mathrm{GT}} = 1$ (h) scenarios. (i-k) Inferred against ground-truth α indices in multi-MIC scenarios with $m_C = 1$ (i), $m_C = 2$ (j), $m_C = 3$ (k); for each MIC, $\mathbf{w}_X$ is drawn to yield randomly-distributed ground-truth α profiles. Each dot is one channel from one simulation; colors indicate the four geometric components (α_{ss}: green, α_{ns}: blue, α_{sn}: brown, α_{nn}: magenta). Black line: identity. We ran 10 simulations per scenario in (a-h) and 30 simulations per m_C in (i-k). $n_X = n_Y = 20$, $n_Z = 10$, $m_A = 2$, $m_B = m_D = 0$, $N = 1000$ in all simulations.

bioRxiv preprint doi: https://doi.org/10.64898/2026.05.14.725145; this version posted May 18, 2026. The copyright holder for this preprint (which was not certified by peer review) is the author/funder, who has granted bioRxiv a license to display the preprint in perpetuity. It is made available under a CC-BY 4.0 International license.

Y multiplicatively through the MIC, with $\mathbf{w}_X = [0\ 1]^\top \cdot \gamma$, $\mathbf{w}_Y = \mathbf{w}_Z = 1$. $m_B = m_D = 0$ throughout. Modulation strength γ is swept from 0 to 1.2 in steps of 0.2 over 5 independent repeats. AIS EV is additionally evaluated fitting the model on X_1 and X_2 separately, to make explicit which source component is captured by the AIS.

High-dimensional rank recovery; Fig. 2(f, g). We then moved to a high-dimensional setting with $n_Z = n_X = n_Y = 10$ and $N = 2000$. Ground-truth ranks m_A and m_C are swept jointly from 0 to 5 (with $m_B = m_D = 0$ and Z, X drawn as independent Gaussians), with 5 independent simulations per rank pair, resulting in 30 simulations per marginal rank. Rank selection is followed by a permutation significance test on held-out predictions (1000 shuffles; $p < 0.05$ required to retain non-zero rank).

Data efficiency; Fig. 2h-i and Fig. S2. We assessed data efficiency by sweeping dimension $d := n_X = n_Y = n_Z \in \{10, 15, 20\}$ and sample size N over 10 log-spaced values in [20, 20000], with $m_A = 2$, $m_C = 1$, $m_B = m_D = 0$, all factor matrices drawn uniformly on the unit sphere, and 30 simulations per (d, N). At each (d, N) the rank-selected fit (Algorithm 1) is compared against a full-rank counterpart in which the modulation tensor or AIS are fit without rank constraint. We report cross-validated AIS EV and EV_{mod} (Fig. 2h-i for $d = 10$; Fig. S2a-c, f-h, all d). To probe scaling, both EVs are then normalized by the ground-truth EV computed on each test fold from the true A, W_X, W_Y, W_Z, and replotted against N rescaled by parameter count: N/d for low-rank fits, N/d^2 for full-rank AIS, N/d^3 for full-rank MICs (Fig. S2d-e, i-j).

Geometric decomposition, Fig. 3. We validated the geometric decomposition with $n_X = 50$, $n_Y = 5$, $n_Z = 1$, $m_A = 2$, $m_C = 1$, $N = 1000$, and 30 simulations per scenario. The source MIC weight $\mathbf{w}_X$ is drawn at a fixed angle θ_X to $\text{span}(U_A)$, while the target weight $\mathbf{w}_Y$ is always drawn within $\text{span}(V_A)$ ($\theta_Y = 0$). Two ground-truth geometries are tested: $\theta_X = 0$, corresponding to $\alpha_{ss}^{\text{GT}} = 1$ with all other components zero (Fig. 3b-d), and $\theta_X = \arccos\sqrt{3/4}$, corresponding to $\alpha_{ss}^{\text{GT}} = 3/4$, $\alpha_{ns}^{\text{GT}} = 1/4$ (Fig. 3e-g). The shuffle null for the α indices is constructed from 1000 random draws of $(\mathbf{w}_X, \mathbf{w}_Y)$ conditioned on the fitted $\hat{A}$, and constrained-fit EV_{mod} is evaluated for all combinations of source and target geometric constraints.

Models sweep with pipeline ablations; Fig. S1. We performed an exhaustive sweep over all $2^4 = 16$ binary rank combinations $(m_A, m_B, m_C, m_D \in \{0, 1\})$ with $n_Z = n_X = n_Y = 5$, $N = 1000$, and 15 simulations per scenario. For each scenario, two subspace alignment conditions are tested: *rand*, in which all weight vectors are drawn independently from the unit sphere; and *corr*, applied only when at least two of m_B, m_C, m_D are non-zero, in which the relevant weight vectors of all non-zero terms are set equal to a shared unit vector per space, making all interaction subspaces perfectly co-aligned. The full $2 \times 2 \times 2$ factorial of pipeline conditions - (regress out Z) $\times$ (permutation significance test) $\times$ (rand/corr) - is evaluated, yielding panels (b-i) of Fig. S1. The significance test uses 1000 permutation shuffles.

Non-Gaussian emission models, Fig. S3. This simulation departs from the others only in the observation step. To test robustness to emission-model misspecification, we used the generative process of Eq. S38 as a latent rate-level model and observed counts through a non-linear emission. Specifically, the latent variables $\mathbf{X}, \mathbf{Y}$ produced by Eq. S38 model, with W_Y rescaled by a modulation-strength scalar γ, are interpreted as log-rates after a constant baseline shift: $\boldsymbol{\eta}_X = h\,\mathbf{1}_{n_X}^\top + \mathbf{X}$ and $\boldsymbol{\eta}_Y = \mathbf{1}_{n_Y}^\top + \mathbf{Y}$, with $h = 2$ and entrywise clipping $|\eta| \leq 4$ for numerical stability. Three emission models were compared:

- *Gaussian benchmark*: $\mathbf{X}^{\text{obs}} = \mathbf{X}$, $\mathbf{Y}^{\text{obs}} = \mathbf{Y}$ (the latent rate-level variables, recovering the standard setup);

bioRxiv preprint doi: https://doi.org/10.64898/2026.05.14.725145; this version posted May 18, 2026. The copyright holder for this preprint (which was not certified by peer review) is the author/funder, who has granted bioRxiv a license to display the preprint in perpetuity. It is made available under a CC-BY 4.0 International license.

- *Poisson*: $X_{ti}^{\text{obs}} \sim \text{Poisson}(e^{\eta_{X,ti}})$, $Y_{ti}^{\text{obs}} \sim \text{Poisson}(e^{\eta_{Y,ti}})$, independently across entries;
- *zero-inflated Poisson* (zero-inflation probability $p = 0.5$): $X_{ti}^{\text{obs}} = D_{ti}^{X}\,\tilde{X}_{ti}$ with $\tilde{X}_{ti} \sim \text{Poisson}(e^{\eta_{X,ti}})$ and $D_{ti}^{X} \sim \text{Bernoulli}(1-p)$, analogously for Y.

We swept $\gamma \in [0, 3]$ in steps of 0.25, with 50 simulations per γ. Across conditions, $n_X = n_Y = n_Z = 5$, $N = 10^4$, $m_A = m_B = m_C = 1$, $m_D = 0$. The MICs pipeline – which assumes Gaussian observations – was applied without modification, fitting at the ground-truth ranks. We report EV_{mod} and the mean principal angle between fitted and ground-truth $(\mathbf{w}_X, \mathbf{w}_Y, \mathbf{w}_Z)$ as a function of γ. EV_{mod} increased monotonically with γ in all three conditions Fig. S3b,d,f), and the mean angle error decreased toward zero as γ grew, indicating reliable recovery of the ground-truth MIC geometry despite emission-model misspecification. As expected, absolute EV_{mod} was attenuated under count noise relative to the Gaussian benchmark, with zero-inflated Poisson showing the largest attenuation.

Comparison with Z-splitting baseline; Fig. S4. To benchmark MICs against the Z-splitting baseline, we simulated data with $n_Z = n_Y = 5$, $n_X \in \{1, 3, 5, 7, 9\}$, $N = 500$, $m_A = 2$, $m_C = 1$, and $m_B = m_D = 0$, running 100 independent simulations per n_X value. For the splitting baseline, $\mathbf{Z}$ is projected onto its first principal component and samples are partitioned into $N_{\text{bins}} \in \{1, \ldots, 15\}$ equipopulated quantile bins; a separate rank-selected AIS is fit within each bin using the same cross-validation partition as MICs, and modulatory EV is defined as the difference between the mean within-bin AIS EV and the EV of a single AIS fit pooled across all samples. For $n_Z > 1$, this projection discards variance in $\mathbf{Z}$ beyond its leading principal component. MIC modulatory EV and the peak splitting EV (maximized over N_{bins}) are then compared as a function of n_X.

Single-component and multi-MIC geometries, Fig. S5. We extended the geometric-decomposition validation in two directions. Across all panels, $n_X = n_Y = 20$, $n_Z = 10$, $m_A = 2$, $m_B = m_D = 0$, and $N = 1000$. Panels (a-h) cover the four pure single-component scenarios $\alpha_k^{\text{GT}} = 1$, $k \in \{ss, ns, sn, nn\}$, with $m_C = 1$ and 10 simulations per scenario: $\mathbf{w}_X$ is drawn uniformly on the unit sphere of either $\text{span}(U_A)$ (s on the source side) or its orthogonal complement (n), and analogously for $\mathbf{w}_Y$ with respect to $\text{span}(V_A)$; $\mathbf{w}_Z$ is drawn uniformly on the unit sphere of $\mathbb{R}^{n_Z}$. For each fitted MIC, null-subtracted α indices use 1000 random-rotation shuffles, and constrained-fit EV_{mod} is evaluated under all four combinations of source and target geometric constraints (no/source/target/both). Panels (i-k) cover multi-MIC scenarios with $m_C \in \{1, 2, 3\}$ and 30 simulations per m_C: for each channel, angles θ_X, θ_Y are drawn independently and uniformly from $[0, \pi/2]$, and $\mathbf{w}_X$, $\mathbf{w}_Y$ are constructed at these angles to $\text{span}(U_A)$ and $\text{span}(V_A)$ respectively, yielding randomly distributed ground-truth indices $\alpha_{ss}^{\text{GT}} = \cos^2\theta_X \cos^2\theta_Y$, $\alpha_{ns}^{\text{GT}} = \sin^2\theta_X \cos^2\theta_Y$, $\alpha_{sn}^{\text{GT}} = \cos^2\theta_X \sin^2\theta_Y$, $\alpha_{nn}^{\text{GT}} = \sin^2\theta_X \sin^2\theta_Y$.

A.10 Statistical testing of modulatory and AIS EV

Motivation. The 1-SEM rule used for rank selection (Algorithm 1; [16, 43]) is a heuristic that can yield spurious AIS or modulatory EV in a fraction of cases when the ground-truth m_A or m_C is zero. Across the $m_A^{\text{GT}} = 0$ or $m_C^{\text{GT}} = 0$ scenarios of Fig. S1 (*rand*, Step 1 on), the rule alone retained $m_A \geq 1$ and $m_C \geq 1$ in 18 % and 34 % of simulations, respectively. We therefore complement rank selection with a permutation test, applied hierarchically to AIS ($m_A > 0$) and to modulation ($m_C > 0$), that decides on held-out data whether each candidate term predicts Y better than expected under a null in which it is temporally unrelated to the test-set residuals it is meant to explain.

Test construction. We split the dataset into two non-overlapping halves (first and second half of samples). For each split, the MICs pipeline (rank selection, ridge regularization, and ALS)

bioRxiv preprint doi: https://doi.org/10.64898/2026.05.14.725145; this version posted May 18, 2026. The copyright holder for this preprint (which was not certified by peer review) is the author/funder, who has granted bioRxiv a license to display the preprint in perpetuity. It is made available under a CC-BY 4.0 International license.

is fit on one half and evaluated on the other, so that all fitting and hyperparameter selection are confined to the training half. On the held-out half we form three predictions of Y of increasing complexity,

$$\hat{Y}_B = Z_{\text{test}} B, \qquad \hat{Y}_{AB} = \hat{Y}_B + X'_{\text{test}} A, \qquad \hat{Y}_{\text{full}} = \hat{Y}_{AB} + \left[(X'_{\text{test}} W_X) \odot (Z_{\text{test}} W_Z)\right] W_Y^\top,$$

where $X' = X - ZD$ uses the regression D fit in Step 1 of Algorithm 1. The observed held-out EV gains

$$\Delta\text{EV}_{AIS} = R^2(\hat{Y}_{AB}) - R^2(\hat{Y}_B), \qquad \Delta\text{EV}_{mod} = R^2(\hat{Y}_{\text{full}}) - R^2(\hat{Y}_{AB}),$$

quantify the variance in Y_{test} captured by AIS and by modulation, respectively. $R^2(\hat{Y})$ denotes the coefficient of determination of real Y from prediction $\hat{Y}$ compared against Y training mean.

To test significance of ΔEV_{mod}, we shuffle in time the predicted modulation component $\hat{\Delta}_{mod} = \hat{Y}_{\text{full}} - \hat{Y}_{AB}$ $n_{\text{shuf}} = 1000$ times. For time series, we apply random circular shifts uniformly drawn from $[\lfloor N_{\text{test}}/4 \rfloor, \lfloor 3N_{\text{test}}/4 \rfloor]$, where N_{test} denotes the number of test samples. For i.i.d. samples from simulations, we randomly permute $\hat{\Delta}_{mod}$ across all test samples. We then recompute $\Delta\text{EV}_{mod}^{shuff} = R^2(\hat{Y}_{AB} + \hat{\Delta}_{mod}^{shuff}) - R^2(\hat{Y}_{AB})$ on each shuffle. Circular shifting destroys the temporal alignment between $\hat{\Delta}_{mod}$ and the residual $Y_{\text{test}} - \hat{Y}_{AB}$ while preserving the marginal distribution and autocorrelation of $\hat{\Delta}_{mod}$, so that adding back a misaligned $\hat{\Delta}_{mod}^{shuff}$ to $\hat{Y}_{AB}$ generally inflates the sum of squared errors and yields a null statistic with negative mean. A genuine modulatory contribution should therefore exceed essentially all shuffles. Null EV are averaged across the two splits, and a one-sided p-value is computed empirically, comparing ΔEV_{mod} to the $\Delta\text{EV}_{mod}^{shuff}$ distribution. ΔEV_{AIS} significance is tested identically with $\hat{\Delta}_{AIS} = X'_{\text{test}} A$ in place of $\hat{\Delta}_{mod}$ and $\hat{Y}_B$ in place of $\hat{Y}_{AB}$. A non-zero rank is retained only if $p < 0.05$; otherwise the corresponding term is fixed to zero in all downstream analyses (geometry indices, constrained-fit comparisons).

Testing m_A and m_C with this procedure reduces the average false-positive rate at $m_C^{\text{GT}} = 0$ from 34 % to 0 % across the 16 scenarios of Fig. S1 while preserving a true-positive rate of 100 % at $m_C^{\text{GT}} = 1$ (vs. 100 % without the test); analogous values for m_A: 18 %, 4 %, 100 %, 100 %.

A.11 Details and further analyses of ACA-VIP data

Experimental procedures and preprocessing. We collected simultaneous dual-color two-photon calcium imaging of ACA axonal projections in V1 and V1 VIP interneurons in head-fixed mice during viewing of visual stimuli, while continuously tracking pupil diameter and running speed. All procedures performed in this study were in accordance with the Massachusetts Institute of Technology's Animal Care and Use Committee and the Guide for the Care and Use of Laboratory Animals published by the National Institutes of Health. VIP-Cre mice (Jackson stock no: 031628, RRID:IMSR_JAX:031628) were anesthetized with isoflurane (1.5%) and given preemptive analgesia (extended release buprenex, 1mg/kg, and meloxicam, 5mg/kg, s.c.). After being placed in a stereotaxic frame and prepared for surgery, a small craniotomy was made above the ACA (AP: +1, ML: -0.3, DV: -0.9) and 0.3 ul of AAV1-hSyn-axon-GCamP6s [59] was injected into the ACA with a micropipette. A larger craniotomy (3mm) was made above the V1 and 4 small injection of 0.015ul AAV9-DIO-jRGECO1a [60] was made into the V1 before placing the cranial window and securing it on the skull with dental cement. Visual stimuli were drifting gratings (8 directions × 3 contrasts, 10 trials per condition; 240 trials/session) or natural movies (5 movies × 3 contrasts, 10 trials per condition; 150 trials/session). Moreover, 25 of 28 sessions included 10 mild airpuffs (compressed air at 40 psi for 0.3 s) per session in alternating trial blocks to evoke arousal events. Frames were acquired at 33 Hz and every other frame was averaged, yielding an effective sampling rate of ∼16.5 Hz (∼24,600 samples/session, ∼25 min). Behavioral, optical, and stimulus-design conventions otherwise followed [49]. Calcium traces were neuropil-corrected as $F_{\text{ROI}} = F_{\text{ROI,raw}} - 0.7\, F_{\text{neuropil}}$. To obtain single-axon traces, ACA boutons were grouped into putative single-axon clusters by hierarchical clustering on pairwise

bioRxiv preprint doi: https://doi.org/10.64898/2026.05.14.725145; this version posted May 18, 2026. The copyright holder for this preprint (which was not certified by peer review) is the author/funder, who has granted bioRxiv a license to display the preprint in perpetuity. It is made available under a CC-BY 4.0 International license.

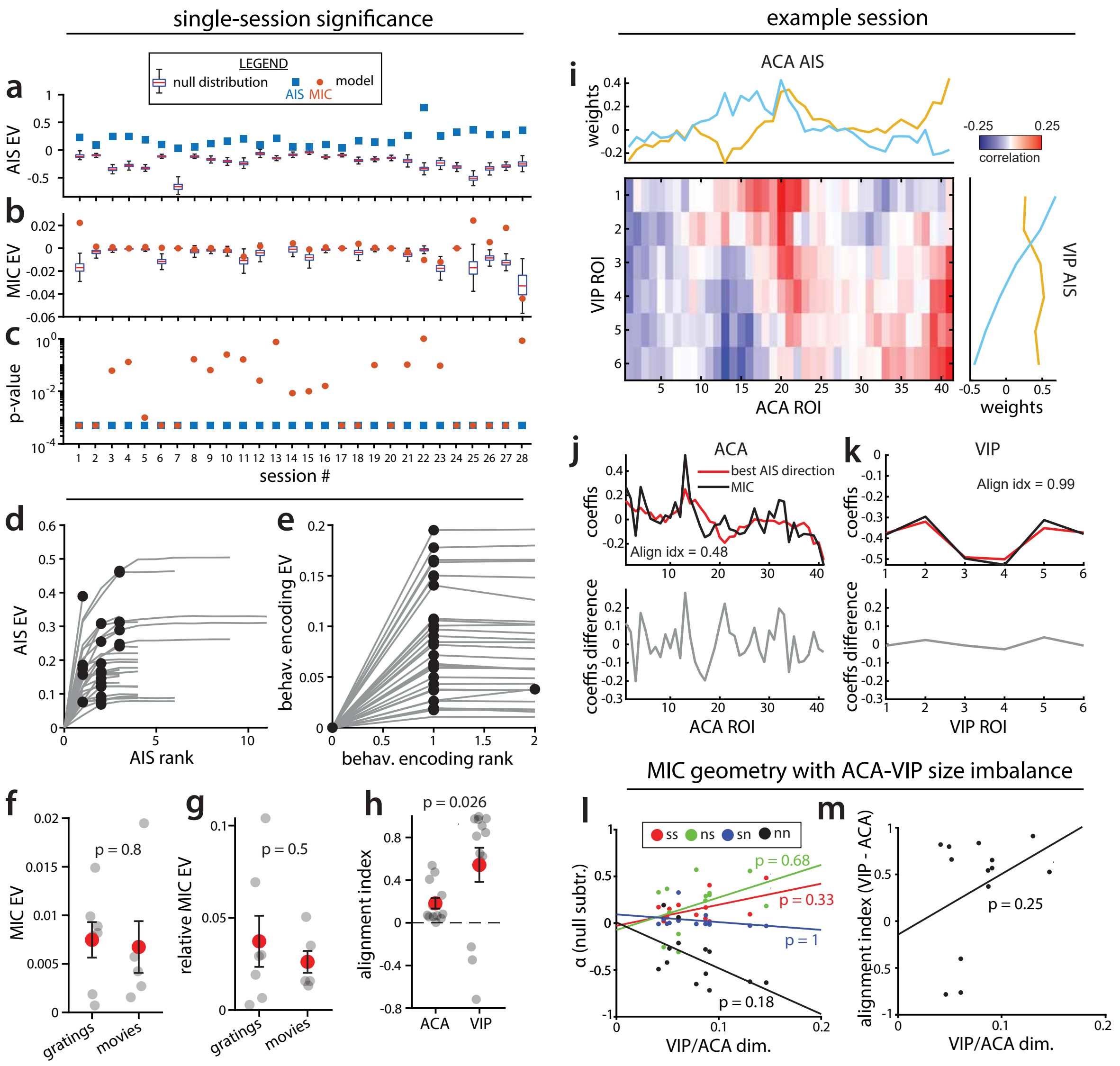

Figure S6: **Additional analyses of ACA-VIP recordings.** (a) Single-session AIS EV significance against a shuffle null (see App. A.10). Boxes: null distribution median and IQR; whiskers: 1.5×IQR; dots: mean held-out AIS EV. (b) Same as (a) but for EV MIC. (c) Single-session empirical AIS EV and MIC EV p-values. (d) AIS EV as a function of AIS rank; gray lines, individual session; black dots, single-session selected rank. (e) Same as (d) for behavioral state encoding EV (term B). (f) Comparison of MIC EV in sessions with different visual stimuli (drifting gratings vs. natural movies). Gray circles: individual sessions; red circles: mean ± SEM across sessions. Only sessions with significant (shuffle test $p < 0.05$, see (a)) are shown. (g) Same as (f), but for the ratio of MIC EV to AIS EV. (h) MIC-AIS alignment index for ACA weights (left) and VIP weights (right) across sessions. (i) AIS low-rank decomposition for one example session ($m_A = 2$). Heatmap: ACA-VIP cross-correlation matrix after regressing out behavioral state (Step 1, Alg. 1). Top: ACA AIS weights. Right: VIP AIS weights. For visualization purposes, ACA and VIP ROIs were sorted using optimal leafs order of hierarchical clustering (Euclidean distance, Ward linkage). (j) MIC alignment with AIS for ACA ($m_C = 1$). Top: MIC weights (black) and their best approximation within the AIS (red). Bottom: residual difference. Alignment index reported in panel. (k) Same as (f) for VIP. (l) Null-subtracted geometry indices α_{ss} (red), α_{ns} (green), α_{sn} (blue), and α_{nn} (black) as a function of the VIP:ACA population size ratio. Lines: best linear fits. (m) Same as (l), but for the difference between VIP and ACA MIC-AIS alignment index. P-values show two-tailed t-test in (f-g), one-tailed paired t-test in (h), and Bonferroni corrected Pearson's correlation tests in (i-j).

bioRxiv preprint doi: https://doi.org/10.64898/2026.05.14.725145; this version posted May 18, 2026. The copyright holder for this preprint (which was not certified by peer review) is the author/funder, who has granted bioRxiv a license to display the preprint in perpetuity. It is made available under a CC-BY 4.0 International license.

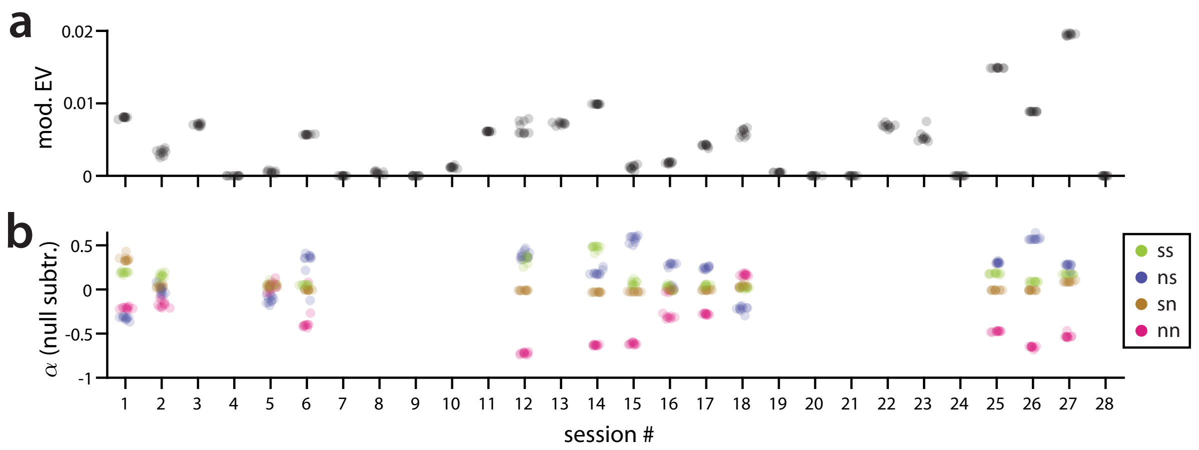

Figure S7: **Robustness of ACA-VIP analyses to ALS random initialization.** For each of the 28 sessions, MICs were re-fitted 10 times with independently drawn random ALS initializations, holding all per-session ranks (m_A, m_B, m_C, m_D) fixed at the values selected in the main text. **(a)** $\mathrm{EV}_{\mathrm{mod}}$ for each session and ALS seed (one dot per seed). **(b)** Null-subtracted geometry indices $\tilde{\alpha}_{ss}$ (green), $\tilde{\alpha}_{ns}$ (blue), $\tilde{\alpha}_{sn}$ (brown), $\tilde{\alpha}_{nn}$ (pink) for the 13 sessions with significant $\mathrm{EV}_{\mathrm{mod}}$, shown for each seed; geometry was not interpreted for sessions without significant modulation.

temporal correlations, and within-cluster traces were averaged with weights proportional to ROI signal-to-noise ratio. All ACA-axon and VIP-neuron traces were linearly detrended within session prior to analysis. We used 28 sessions (15 movies; 13 gratings) collected across 3 mice, taking as response variables the per-axon and per-neuron calcium traces; samples with any NaN across X, Y, or Z were discarded. Included counts ranged 3-212 for ACA axons and 1-11 for VIP neurons.

MICs fitting and main analyses. We fit the pipeline of Algorithm 1 separately to each session, with $K = 10$ temporally contiguous cross-validation folds to avoid leakage between training and test sets due to temporal autocorrelation. We took ACA axons as source X and VIP neurons as target Y, consistent with anatomical directionality, and defined the modulator $Z \in \mathbb{R}^2$ as the joint, within-session-standardized vector of pupil diameter and wheel running speed. AIS and MICs ranks were capped at $m_A^{\max} = 20$ and $m_C^{\max} = 10$ for computational efficiency. The MIC correlation slope (Fig.4g) was computed per channel by projecting X, Y, Z onto $w_{X,i}$, $w_{Y,i}$, $w_{Z,i}$, partitioning $Zw_{Z,i}$ into 5 equipopulated quantiles, computing the Pearson correlation between $Xw_{X,i}$ and $Yw_{Y,i}$ within each quantile, and taking the OLS slope of these five correlations against quantile index; slopes were averaged across MICs and then across sessions. Geometry was characterized via null-subtracted indices $\tilde{\alpha}_{ss}$, $\tilde{\alpha}_{sn}$, $\tilde{\alpha}_{ns}$, $\tilde{\alpha}_{nn}$ (Fig. 4h, top), where $\tilde{\alpha}_{ns}$ and $\tilde{\alpha}_{sn}$ correspond to recruitment of private ACA (source) and VIP (target) dimensions, respectively. Each index was obtained by subtracting from the fitted α the mean over 1000 random rotations of $w_{X,i}$, $w_{Y,i}$ on their unit spheres (Appendix A.7.3). Constrained-fit $\mathrm{EV}_{\mathrm{mod}}$ (Fig. 4h, bottom) further restricts w_X and/or w_Y to lie within the corresponding AIS following Appendix A.8, with "ACA" and "VIP" denoting the constraint applied to the source and target factor, respectively. Single-session significance of $\mathrm{EV}_{\mathrm{mod}}$ and EV_A was assessed via the residual-shuffle permutation test of Appendix A.10 with $n_{\mathrm{shuf}} = 1000$ shuffles per session (Fig. S6a-c).

Additional analyses. To visualize the MIC-AIS geometry in one example session (Fig. S6i), we computed the best approximation of each MIC factor within the corresponding AIS as $\hat{w}_X = P_X w_X$ and $\hat{w}_Y = P_Y w_Y$, with P_X, P_Y the orthogonal projectors onto the source and target AIS. To compare alignment across animals (Fig. S6h), we summarized per-animal MIC-AIS

bioRxiv preprint doi: https://doi.org/10.64898/2026.05.14.725145; this version posted May 18, 2026. The copyright holder for this preprint (which was not certified by peer review) is the author/funder, who has granted bioRxiv a license to display the preprint in perpetuity. It is made available under a CC-BY 4.0 International license.

alignment by a normalized index that rescales the observed squared cosine by chance alignment,

$$\text{align} \;=\; \frac{\cos^2\theta_{\text{obs}} - \langle\cos^2\theta_{\text{shuff}}\rangle}{1 - \langle\cos^2\theta_{\text{shuff}}\rangle}, \tag{S39}$$

where $\cos^2\theta_{\text{obs}} = \|P\,w\|^2/\|w\|^2$ for the source ($w = w_X$, $P = P_X$) or target ($w = w_Y$, $P = P_Y$) factor (see Appendix A.7.2), and $\langle\cos^2\theta_{\text{shuff}}\rangle$ is the same quantity averaged over uniform random samples of w on the unit sphere, matched in dimension and AIS rank. align = 0 corresponds to chance alignment given the dimensionality; align = 1 to a fitted MIC lying entirely within the AIS. We used a one-tailed test for this analysis under the hypothesis - motivated by geometry analyses in Fig 4 - that VIP MICs are more aligned to the AIS, compared to ACA. To rule out that the asymmetric alignment between ACA and VIP factors was driven by the typical $n_{\text{ACA}} : n_{\text{VIP}} \approx 15$ population-size imbalance of sessions with significant EV_{mod}, we regressed the per-session ACA-vs-VIP alignment difference and the four null-subtracted α indices against the ratio $n_{\text{VIP}}/n_{\text{ACA}}$. Furthermore, to test whether airpuff-evoked arousal events drove the recovered modulation, we ran a control analysis recomputing EV_{mod} after excluding the 10 s window following each airpuff onset (not shown); EV_{mod} did not differ significantly from the full-session value, suggesting that modulation is driven by endogenous fluctuations in behavioral state rather than puff-evoked transients. Consistently, 2 of the 3 puff-free sessions were among the 13 sessions with significant EV_{mod}. Finally, to assess robustness of MIC fits to the non-convexity of the ALS objective (Appendix A.5), we re-fitted MICs on each session with 10 independent random ALS initializations, holding the per-session ranks (m_A, m_B, m_C, m_D) fixed at the values selected in the main text. For each seed we recomputed EV_{mod} for all sessions and the null-subtracted α indices for the 13 sessions with significant EV_{mod} (Fig. S7). Both EV_{mod} and the null-subtracted α indices were tightly clustered across seeds, indicating that the ACA-VIP findings are stable under the non-convexity of the ALS objective.

A.12 Computational resources

All simulations and real data analyses ran on a workstation with a 24 cores Intel i9-14900K processor and 192 GB of RAM, running Windows 11 and MATLAB R2025b, with parallelization over 20 workers (MATLAB Parallel Computing Toolbox). The pipeline additionally requires the Statistics and Machine Learning Toolbox.

Simulations in Figure 2a-e ran in approximately 10 minutes. All other simulations in Figures 2, 3, S1, S2, S3, S4, and S5 ran in less than one day. Real data analyses in Figures 4 and S6 also completed in under one day on the same machine, except for the residual-permutation test and the constrained model fitting, which took approximately two days.
